# Characterization of the O-Glycoproteome of Tannerella forsythia

**DOI:** 10.1128/mSphere.00649-21

**Published:** 2021-09-15

**Authors:** Paul D. Veith, Nichollas E. Scott, Eric C. Reynolds

**Affiliations:** a Oral Health Cooperative Research Centre, Melbourne Dental School, Bio21 Institute, The University of Melbourne, Victoria, Australia; b Department of Microbiology and Immunology, University of Melbourne at the Peter Doherty Institute for Infection and Immunity, Melbourne, Australia; University of Iowa

**Keywords:** *Tannerella forsythia*, O-glycosylation, glycoproteins, proteome, trifluoromethanesulfonic acid, glycans, mass spectrometry

## Abstract

Tannerella forsythia is a Gram-negative oral pathogen known to possess an O-glycosylation system responsible for targeting multiple proteins associated with virulence at the three-residue motif (D)(S/T)(A/I/L/V/M/T). Multiple proteins have been identified to be decorated with a decasaccharide glycan composed of a poorly defined core plus a partially characterized species-specific section. To date, glycosylation studies have focused mainly on the two S-layer glycoproteins, TfsA and TfsB, so the true extent of glycosylation within this species has not been fully explored. In the present study, we characterize the glycoproteome of *T. forsythia* by employing FAIMS-based glycopeptide enrichment of a cell membrane fraction. We demonstrate that at least 13 glycans are utilized within the *T. forsythia* glycoproteome, varying with respect to the presence of the three terminal sugars and the presence of fucose and digitoxose residues at the reducing end. To improve the localization of glycosylation events and enhance the detection of glycopeptides, we utilized trifluoromethanesulfonic acid treatment to allow the selective chemical cleavage of glycans. Reducing the chemical complexity of glycopeptides dramatically improved the number of glycopeptides identified and our ability to localize glycosylation sites by ETD fragmentation, leading to the identification of 312 putative glycosylation sites in 145 glycoproteins. Glycosylation site analysis revealed that glycosylation occurs on a much broader motif than initially reported, with glycosylation found at (D)(S/T)(A/I/L/V/M/T/S/C/G/F). The prevalence of this broader glycosylation motif in the genome suggests the existence of hundreds of potential O-glycoproteins in this organism.

**IMPORTANCE** Tannerella forsythia is an oral pathogen associated with severe forms of periodontal disease characterized by destruction of the tooth’s supporting tissues, including the bone. The bacterium releases a variety of proteins associated with virulence on the surface of outer membrane vesicles. There is evidence that these proteins are modified by glycosylation, and this modification is essential for virulence in producing disease. We have utilized novel techniques coupled with mass spectrometry to identify over 13 glycans and 312 putative glycosylation sites in 145 glycoproteins within *T. forsythia*. Glycosylation site analysis revealed that this modification occurs on a much broader motif than initially reported such that there is a high prevalence of potential glycoproteins in this organism that may help to explain its role in periodontal disease.

## INTRODUCTION

Tannerella forsythia is a Gram-negative, anaerobic, fusiform bacterium associated with periodontitis in humans (1–4). Periodontitis is a chronic inflammatory disease characterized by the destruction of the tooth’s supporting tissues, including bone. Of particular interest in *T. forsythia* biology is the presence of a surface layer (S-layer) composed of two glycoproteins, TfsA and TfsB, that surrounds the cell (5, 6) and is associated with virulence (7, 8). The S-layer also surrounds its outer membrane vesicles (OMVs), which are naturally released into the environment and into the host during periodontal disease (9, 10). TfsA and TfsB are highly glycosylated, and in 2011 it was determined that this glycosylation was achieved by a protein O-glycosylation system (11).

Protein O-glycosylation in *T. forsythia* is related to the system first described in Bacteroides fragilis (12) and then extended to diverse families within the *Bacteroidetes* phylum (13). The glycosylation site motif in B. fragilis was elegantly demonstrated by site-directed mutagenesis to be (D)(S/T)(A/I/L/V/M/T), with the glycan O-linked to the Ser or Thr residue in the 2nd position of the motif (12). The eight identified glycoproteins were all exported to the periplasm or outer membrane (OM), and export was shown to be required for glycosylation to occur, since deletion of a glycoprotein’s signal peptide resulted in retention of the nonglycosylated protein in the cytoplasm. Furthermore, deletion of an essential component of the glycosylation machinery resulted in impaired colonization of mouse intestines, demonstrating the *in vivo* importance of this glycosylation system in virulence (12). Over 1,000 glycoproteins are predicted in B. fragilis based on the presence of the O-glycosylation motif, and a total of 20 glycoproteins have been identified (14). The draft structure of the B. fragilis glycan contains 9 sugars, and it was shown that B. fragilis proteins could be expressed in *T. forsythia* and be decorated with *T. forsythia* glycans and vice versa (15).

**FIG 1:**
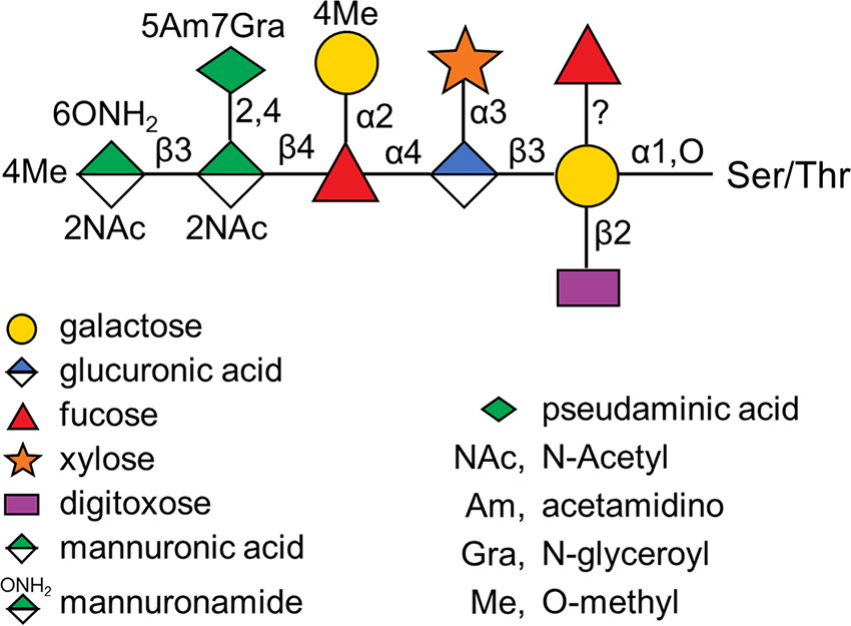
Published structure of *T. forsythia* O-linked glycan. The structure was obtained from previous studies (11, 16) and drawn according to the Symbol Nomenclature for Glycans (SNFG) (30).

In *T. forsythia* to date, 13 glycopeptides have been identified derived from 4 glycoproteins, including TfsA and TfsB, with inferred glycosylation sites consistent with the motif found in B. fragilis (11). The structure of the glycan in strain ATCC 43037 was solved by nuclear magnetic resonance (NMR) and mass spectrometry (MS) methods to contain 10 sugars (11) and was revised with respect to the position of a nonstoichiometric fucose residue in a subsequent publication (16) (Fig. 1). While both the *O*-oligosaccharyl transferase responsible for transferring the glycan to the protein as well as the glycosyltransferases involved in the biosynthesis of the core region of the glycan are unknown, the biosynthetic pathway of the species-specific portion of the glycan has been elucidated and found to involve five glycosyltransferases and two methyltransferases (16). The biosynthesis of the glycan, its variation between strains, and its roles in virulence were recently reviewed (17).

TfsA and TfsB require a functional type IX secretion system (T9SS) for their secretion across the OM to form the S-layer (18, 19). These cargo proteins along with more than 30 others share a conserved C-terminal domain (CTD) signal to enable this secretion (20–22). The signal is cleaved (22) and the C terminus of the mature protein is conjugated to a polysaccharide via a novel linking sugar in a manner analogous to that for P. gingivalis, apparently via the PorU sortase (19, 23, 24). In *T. forsythia*, the linking sugar was identified as a 2-N-glycyl, 3-N-acetylmannuronic acid (24). Hence, TfsA and TfsB along with other cargo proteins are glycosylated by the O-glycosylation system as well as the T9SS.

In this study, we extend the characterization of the O-glycoproteome of *T. forsythia* to include 312 O-glycosylation sites within 145 glycoproteins and document the various glycans that are present. The glycosylation site motif was also extended to include C/S/G/F as additional residues allowed in the third position.

## RESULTS

### Overall identification data.

The membrane-enriched fraction of *T. forsythia* was used for all analyses. Samples with or without partial deglycosylation were subjected to SDS-PAGE, excision of 12 or 16 gel fractions (see Fig. S1 in the supplemental material), in-gel digestion with trypsin, and liquid chromatography-tandem mass spectrometry (LC-MS/MS). These analyses, described in detail below, ultimately led to the identification of 145 glycoproteins from 445 peptide sequences modified by one or more different glycans. These glycopeptides contained 312 predicted O-glycosylation sites (Tables S1 and S2). The most relevant raw outputs of the Byonic and Mascot searches are also provided (Tables S3 and S4).

10.1128/mSphere.00649-21.1 FIG S1 SDS-PAGE of membrane-enriched fractions. Download FIG S1, DOCX file, 0.3 MB. Copyright © 2021 Veith et al. 2021 Veith et al. https://creativecommons.org/licenses/by/4.0/ This content is distributed under the terms of the Creative Commons Attribution 4.0 International license.

10.1128/mSphere.00649-21.5 TABLE S1 List of identified glycopeptides and glycosylation sites. Download Table S1, XLSX file, 0.05 MB. Copyright © 2021 Veith et al. 2021 Veith et al. https://creativecommons.org/licenses/by/4.0/ This content is distributed under the terms of the Creative Commons Attribution 4.0 International license.

### Survey of glycoforms.

**FIG 2:**
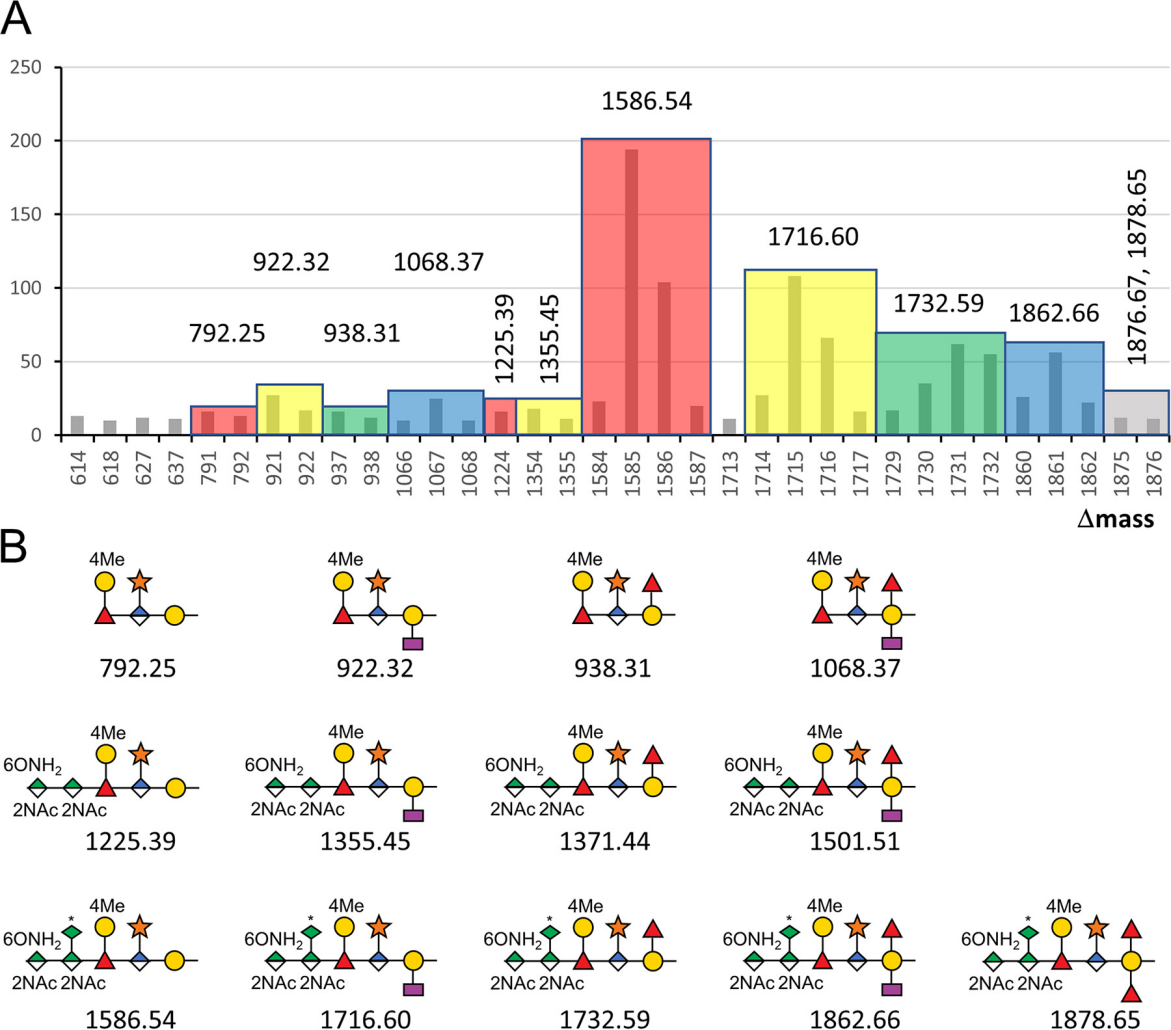
Frequency of glycoforms. (A) The Byonic search results of intact glycoforms showing the frequency of each detected Δmass. Only Δmass values greater than 300 with a frequency of at least 10 are shown. Δmass values were grouped into colored clusters based on exhibiting the same corrected Δmass value as that shown above each group. (B) Assignment of each observed corrected Δmass value to a putative glycan structure. Asterisks indicate that the 7Am5Gra substituents of the pseudaminic acid are not shown. The minor forms at 1,371.44 Da and 1,501.51 Da were observed at lower frequency and are shown in panel B for the sake of completeness.

The MS data produced from the intact (nondeglycosylated) samples were searched with Byonic using a wildcard setting to enable peptides to be identified with any Δmass value. A plot showing the number of identified peptides for frequently observed Δmass values revealed several clusters of potential glycopeptides differing by 1-Da units (Fig. 2A). The clustering is due to the imperfect assignment of the monoisotopic peak by Byonic, and manual checking of a representative number of spectra showed that each cluster was generally due to a single Δmass value corresponding to a single glycoform as shown (Fig. 2A). The data were then searched again with defined modifications instead of wildcards to yield the final data (Tables S1 and S3).

**FIG 3:**
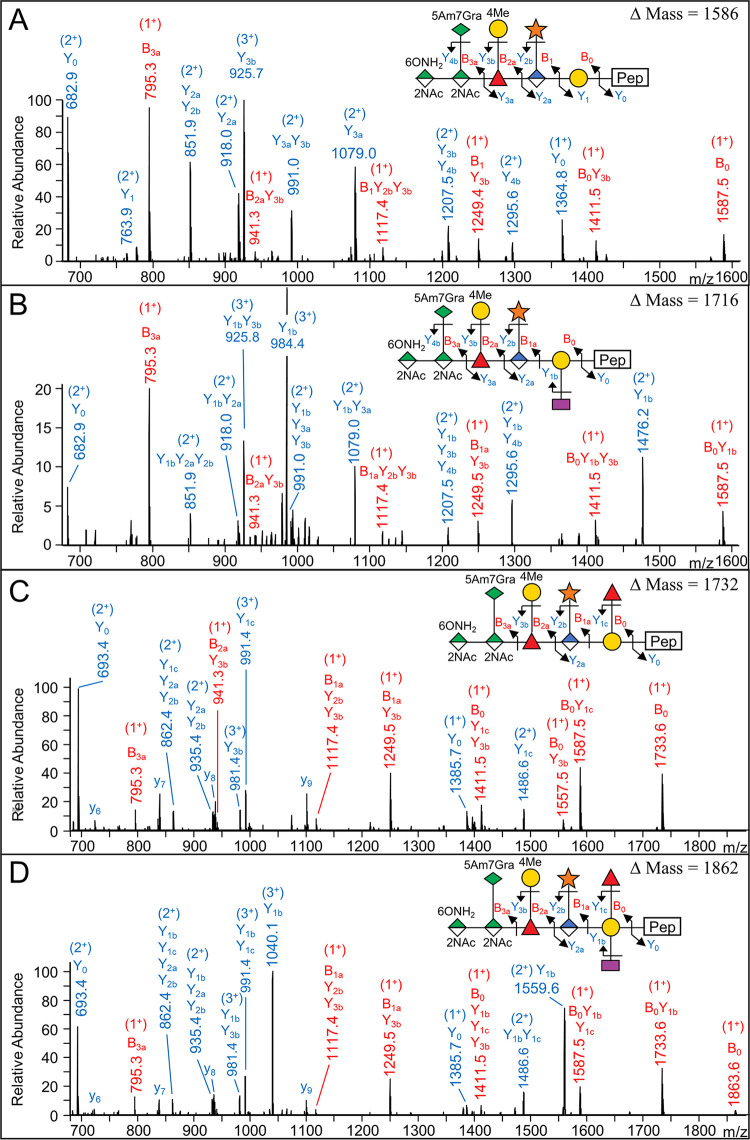
CID spectra of major glycoforms. (A) MS/MS (CID) spectrum of *m/z* = 984.440 (3^+^) matching to the peptide sequence ASNKDTILYVLK (+1,586 Da) from Tanf_03370. (B) MS/MS (CID) spectrum of *m/z* = 1,027.792 (3^+^) matching to the peptide sequence ASNKDTILYVLK (+1,716 Da) from Tanf_03370. (C) MS/MS (CID) spectrum of *m/z* = 1,040.097 (3^+^) matching to the peptide sequence TAIYVDTAYVNR (+1,732 Da) from Tanf_03375. (D) MS/MS (CID) spectrum of *m/z* = 1,083.454 (3^+^) matching to the peptide sequence TAIYVDTAYVNR (+1,862 Da) from Tanf_03375. The fragmentation scheme for each glycan is shown. Peaks labeled with “y” indicate matches to peptide fragments with no glycan additions.

Analysis of the CID spectra of glycopeptides corresponding to the four dominant high-molecular-weight glycoforms allowed the structure of each to be elucidated with reference to the published structure (Fig. 3). The largest form of Δmass 1,862 corresponded to the published full-length glycan minus the methyl group on the N-acetyl mannosaminuronamide (Fig. 3D). The three other major forms differed in the branching at the Gal residue. The most abundant form at Δmass 1,586 had no branching at this point (Fig. 3A), while Δmass 1,716 and 1,732 had a single Dig or Fuc residue, respectively (Fig. 3B and C). These same four branching variants were also observed without the three terminal sugars, with Δmass values of 792, 922, 938, and 1,068, respectively (Fig. 2B). The glycoforms of intermediate Δmass at 1,225 and 1,355 were found to correspond to the first two branching variants but without the terminal Pse sugar (Fig. 2B). The two larger branching variants at 1,371 and 1,501 were also observed but at lower frequency.

**FIG 4:**
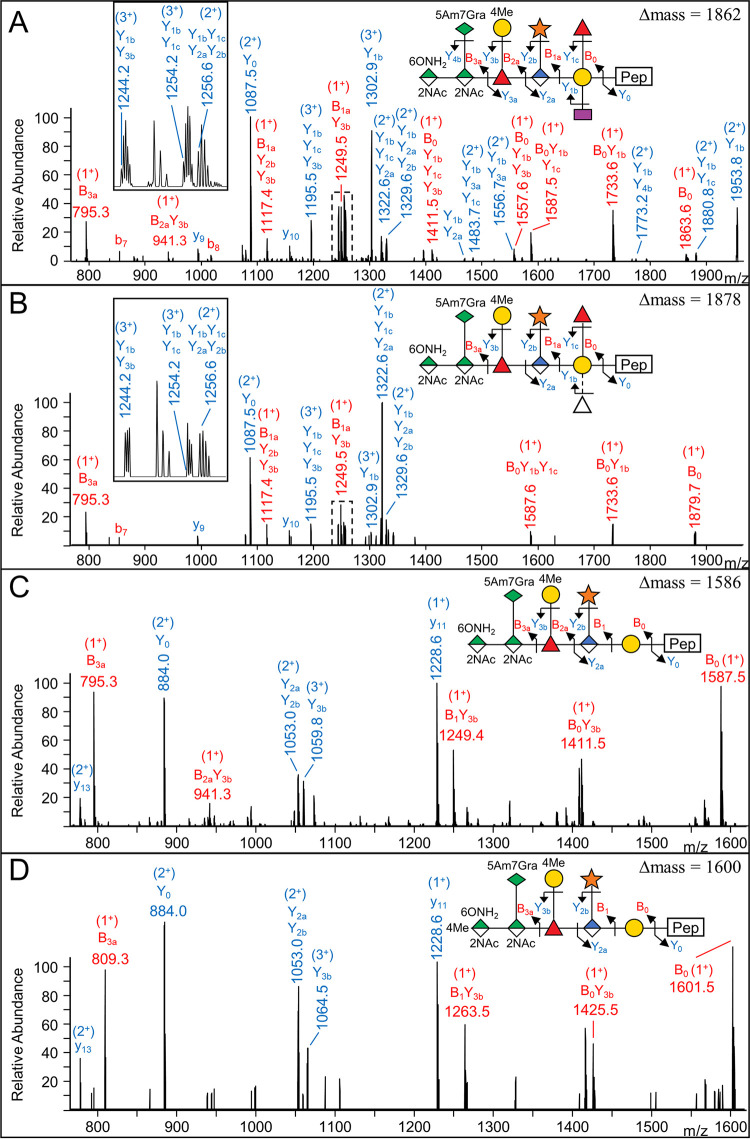
CID spectra of novel glycoform and methylated glycoform. (A) MS/MS (CID) spectrum of *m/z* = 1,346.233 (3^+^) matching to the peptide sequence YLYVDTVYYNSGNSQFLK (+1,862 Da) from Tanf_03375. (B) MS/MS (CID) spectrum of *m/z* = 1,351.562 (3^+^) matching to the peptide sequence YLYVDTVYYNSGNSQFLK (+1,878 Da) from Tanf_03375. Note that 1b and 1c cleavages could not be distinguished in this spectrum. (C) MS/MS (CID) spectrum of *m/z* = 1,118.494 (3^+^) matching to the peptide sequence LVYYPSEDSLVINVR (+1,586 Da) from Tanf_03370. (D) MS/MS (CID) spectrum of *m/z* = 1,123.159 (3^+^) matching to the peptide sequence LVYYPSEDSLVINVR (+1,600 Da) from Tanf_03370. The difference in structure between panels C and D is the methyl group on the mannosaminuronamide. The fragmentation scheme for each glycan is shown. Peaks labeled with “y” or “b” indicate matches to peptide fragments with no glycan additions.

The calculated Δmass of the published full-length glycan is 1,876.67, which was observed for several peptides in the 1,876/1,878 cluster (Fig. 2A); however, most had an observed Δmass of 1,878.65. The CID spectra of glycopeptides with this Δmass revealed an extra deoxyhexose residue and an absence of Dig (Fig. 4A and B). While we could not identify the extra deoxyhexose or its position in the structure, we suggest it may replace the Dig residue. Consistent with this, glycopeptides putatively containing two Dig residues (two dideoxyhexoses) were also identified with a Δmass of 1,846; however, no CID spectra were obtained to confirm the presence of the two Dig residues.

For the published form containing 4-methyl-2-N-acetyl mannosaminuronamide, few CID spectra were obtained. A CID spectrum was obtained for the Δmass 1,600 form and compared to the nonmethylated Δmass 1,586 form (Fig. 4C and D). The low-mass region of the higher-energy C-trap dissociation (HCD) scans also confirmed the presence of the methylated sugar due to the presence of specific ions at *m/z* 231.10 and *m/z* 809.30 (Fig. S2).

10.1128/mSphere.00649-21.2 FIG S2 Low mass region of HCD spectra confirm methylation of N-acetyl mannosaminuronamide. Download FIG S2, DOCX file, 0.7 MB. Copyright © 2021 Veith et al. 2021 Veith et al. https://creativecommons.org/licenses/by/4.0/ This content is distributed under the terms of the Creative Commons Attribution 4.0 International license.

### Partial deglycosylation with TFMS.

**FIG 5:**
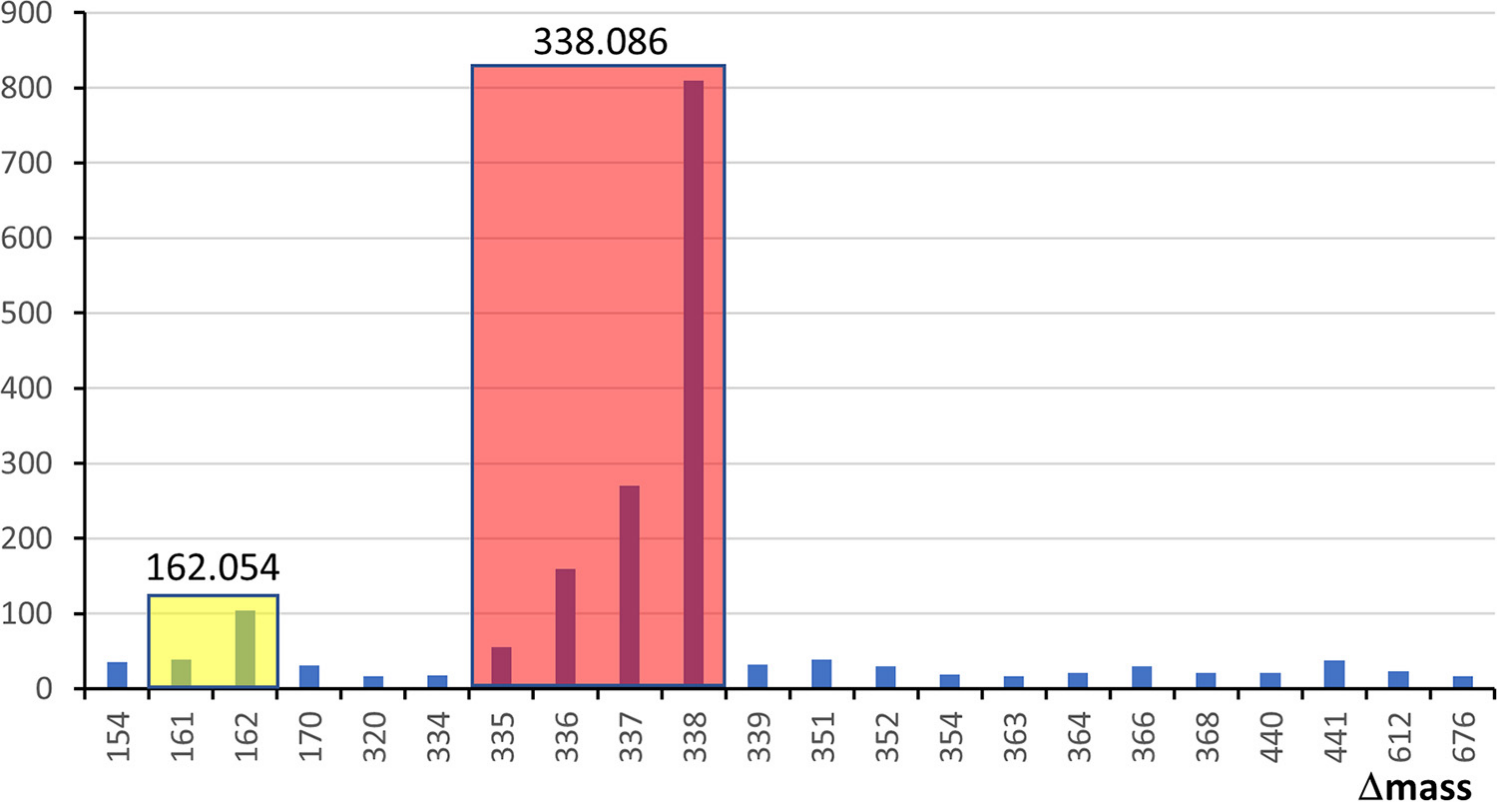
Frequency of acid-cleaved glycoforms. The Byonic search results of acid-cleaved glycoforms showing the frequency of each detected Δmass. Only Δmass values with a frequency of greater than 15 are shown. Only Δmass values associated with peptides containing the O-glycosylation motif were used. Δmass values were grouped into colored clusters based on exhibiting the same corrected Δmass value as shown above each group.

For the samples treated with trifluoromethanesulfonic acid (TFMS), the data were first searched with Byonic in wildcard mode. To specifically examine the effect of acid cleavage on O-glycosylated peptides, identified peptides containing the O-glycosylation motif were extracted from the data, and their frequency was plotted against the observed Δmass values (Fig. 5). The most common Δmass values were a cluster at 335 to 338 Da with an accurate mass of 338.086, consistent with a residual modification of Hex-HexA, and 161 to 162 Da, with an accurate mass of 162.054, consistent with a residual Hex. Therefore, it appeared that the major detectable products of TFMS cleavage of the glycans resulted from clean cleavage of the glycosidic bonds. The data were therefore searched again using Mascot with manually defined modifications based on theoretical cleavage of the published structure. Acid cleavage was found to occur between most sugars, resulting in a wide range of observed Δmass values, but a strong preference was observed for Δmass 338, composed of GlcA-Gal. Peptides with this modification were identified approximately three times as frequently as all other modifications combined and also more frequently than modified peptides from the untreated sample (Tables S1 and S4).

### Identification of glycosylation sites.

**FIG 6:**
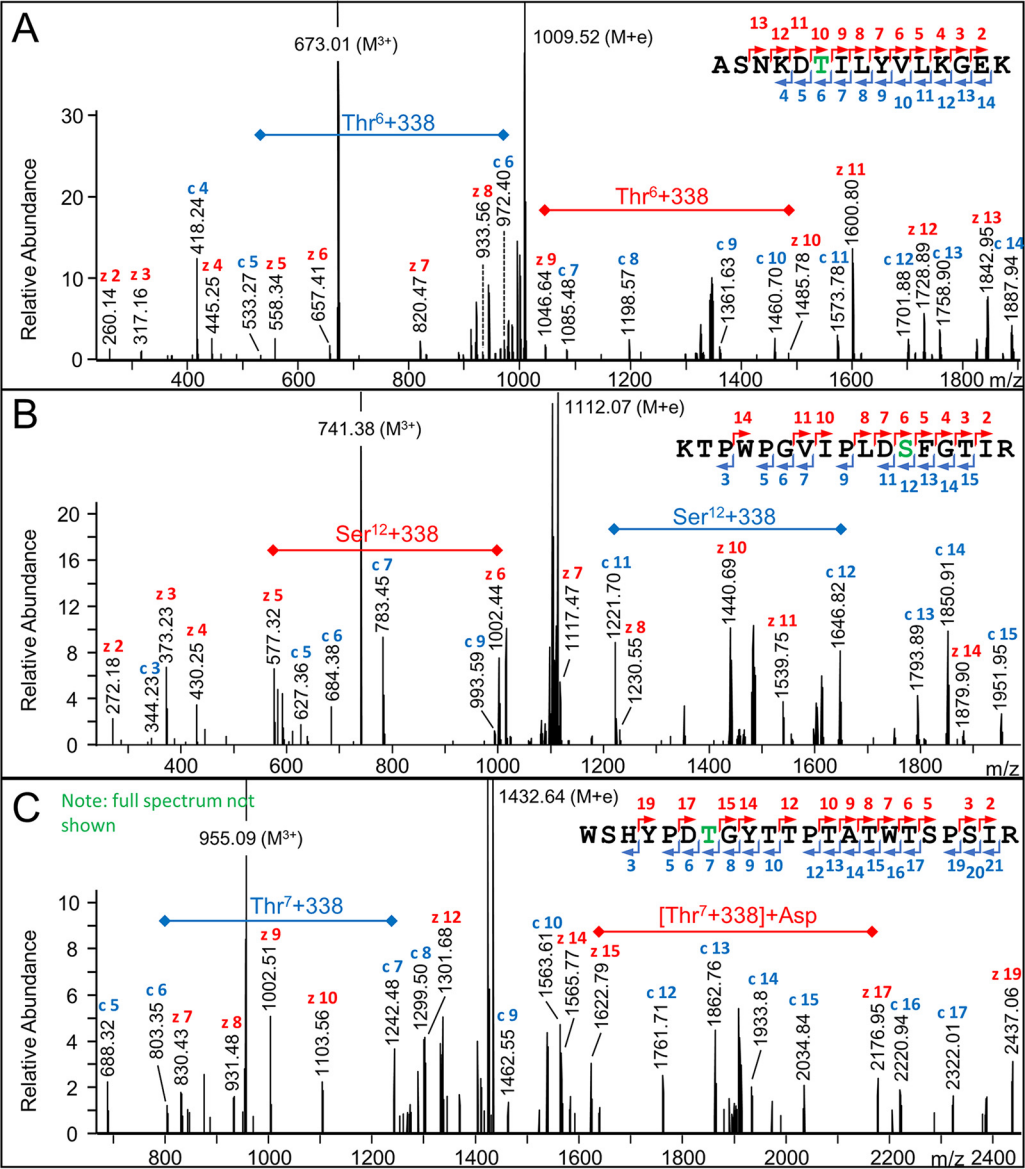
ETD spectra showing localization of glycans after TFMS deglycosylation. (A) ETD spectrum of *m/z* = 673.01 (3^+^) matching to the peptide sequence ASNKDTILYVLKGEK (+338 Da) from Tanf_03370. (B) ETD spectrum of *m/z* = 741.38 (3^+^) matching to the peptide sequence KTPWPGVIPLDSFGTIR (+338 Da) from Tanf_03375. (C) EtHCD spectrum of *m/z* = 955.09 (3^+^) matching to the peptide sequence WSHYPDTGYTTPTATWTSPSIR (+338 Da) from Tanf_011855. For each spectrum, the c-ions and z-ions are single charged and labeled blue and red, respectively, and match the colored numbers in the fragmentation scheme of the peptide sequence. The glycosylation sites are shown in green.

Previously, O-glycosylation in *T. forsythia* and other *Bacteroidetes* was found to occur at Ser or Thr within the motif (D)(S/T)(A/I/L/V/M/T). The data obtained in this study support that glycosylation occurs on a broader range of motifs, with Cys, Ser, Gly, and Phe in the third position (Table S1). To directly confirm glycosylation sites, HCD and electron transfer dissociation (ETD) fragmentation was conducted on selected ions in a repeat set of LC-MS runs of the partially deglycosylated sample. The Byonic “Delta Mod” score provides an indication of the probable glycosylation site when there is more than one possible site in the peptide. Of 35 such unique sites identified with a Delta Mod score of >25, 34 were assigned to the 2nd residue (S/T) of the motif (Table S5), providing strong support for glycosylation at that site. For more accurate assignment, selected MS/MS spectra were examined. For the protein TfsA (Tanf_03370), Thr^603^ was shown to be glycosylated within the peptide ASNKDT*ILYVLKGEK by observing a strong series of both c and z ions across the modification site (Fig. 6A). Similarly, glycosylation sites at the new motifs DS*F and DT*G were also directly demonstrated (Fig. 6B and C). Other examples are shown in Fig. S3, with most providing strong direct evidence of glycosylation at the indicated S/T residue rather than other potential sites.

10.1128/mSphere.00649-21.3 FIG S3 Additional ETD and HCD spectra showing site localization of glycans. Download FIG S3, PDF file, 0.6 MB. Copyright © 2021 Veith et al. 2021 Veith et al. https://creativecommons.org/licenses/by/4.0/ This content is distributed under the terms of the Creative Commons Attribution 4.0 International license.

10.1128/mSphere.00649-21.7 TABLE S3 Byonic output of intact samples. Download Table S3, XLSX file, 2.1 MB. Copyright © 2021 Veith et al. 2021 Veith et al. https://creativecommons.org/licenses/by/4.0/ This content is distributed under the terms of the Creative Commons Attribution 4.0 International license.

10.1128/mSphere.00649-21.8 TABLE S4 Mascot output of TFMS-treated samples. Download Table S4, XLSX file, 2.7 MB. Copyright © 2021 Veith et al. 2021 Veith et al. https://creativecommons.org/licenses/by/4.0/ This content is distributed under the terms of the Creative Commons Attribution 4.0 International license.

10.1128/mSphere.00649-21.9 TABLE S5 Glycosylation site analysis. Download Table S5, XLSX file, 0.02 MB. Copyright © 2021 Veith et al. 2021 Veith et al. https://creativecommons.org/licenses/by/4.0/ This content is distributed under the terms of the Creative Commons Attribution 4.0 International license.

### The proteins identified.

The major class of observed glycoproteins was the T9SS cargo proteins, of which 18 were identified with a total of 120 glycosylation sites, accounting for 38% of the total (Table S2). Outer membrane proteins numbered 53 with 80 glycosylation sites, while proteins localized to the inner membrane and periplasm numbered 12 and 11, respectively. Fifty-one proteins were of uncertain localization. The most heavily glycosylated protein was TfsB (Tanf_03375), with 19/20 predicted glycosylation sites detected. With an average glycan mass of 1,700 Da, this amounts to ∼34 kDa of carbohydrate, excluding the C-terminal polysaccharide associated with the T9SS.

10.1128/mSphere.00649-21.6 TABLE S2 List of identified glycoproteins. Download Table S2, XLSX file, 0.02 MB. Copyright © 2021 Veith et al. 2021 Veith et al. https://creativecommons.org/licenses/by/4.0/ This content is distributed under the terms of the Creative Commons Attribution 4.0 International license.

### Site preference and glycosylation efficiency.

**FIG 7:**
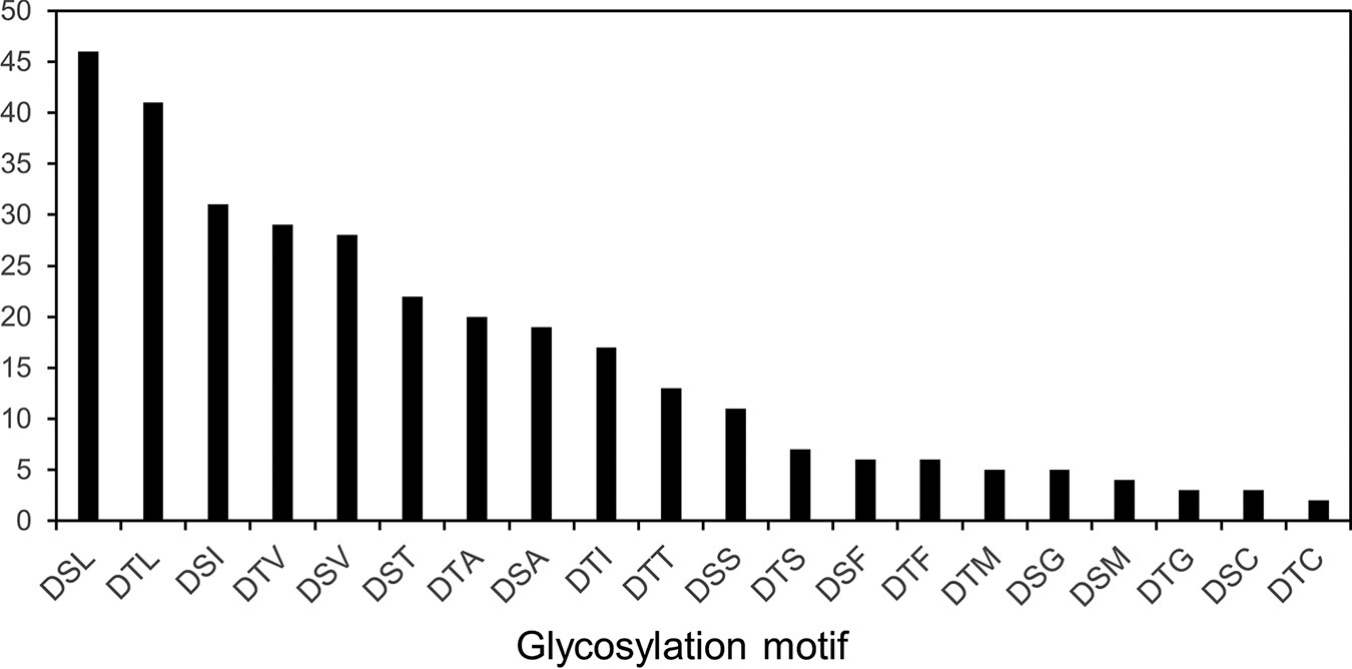
Frequency of detected glycosylation motifs. The number of identified glycosylation sites corresponding to each identified three-residue motif is shown.

In total, 10 different site motifs were identified. Of these, DSL and DTL were most frequently observed, and DTC and DSC were least frequent (Fig. 7). Interestingly, the data appeared to indicate that the various glycoforms were not randomly assigned to sites. Sites modified with Δmass 1,586 were likely to also be modified with Δmass 1,716, whereas Δmass 1,732 exhibited a strong pairing with Δmass 1,862 (Table S1, Fig. S4). Hence, the level of fucose rather than digitose appeared to govern the selectivity. The same fucose selectivity extended to the smaller glycoforms.

10.1128/mSphere.00649-21.4 FIG S4 Correlation of glycoforms according to fucose level. Download FIG S4, DOCX file, 1.1 MB. Copyright © 2021 Veith et al. 2021 Veith et al. https://creativecommons.org/licenses/by/4.0/ This content is distributed under the terms of the Creative Commons Attribution 4.0 International license.

With regard to efficiency, in most cases, the nonmodified counterpart peptides were not identified, and when they were identified, inspection of the extracted ion chromatograms indicated that the peak of the most abundant modified form was usually around 2 orders of magnitude more intense than the unmodified counterpart, suggesting a glycosylation efficiency of >99%. Some exceptions were found, particularly for the new glycosylation sites, such as the Tanf_03375 peptides KLDAASDS*SPAAPK and TPWPGVIPLDS*FGTIR, which exhibited modification efficiencies of ∼1% and ∼70%, respectively. However, this was not a general rule, with other peptides with the DSS or DSF motifs having high efficiencies.

## DISCUSSION

This study is the largest proteomic survey of O-glycosylation in *Bacteroidetes* to date, with the identification of 312 putative O-glycosylation sites within 145 glycoproteins. While only 20 glycoproteins were identified in B. fragilis, 1,021 were predicted based on the presence of the O-glycosylation motif, (D)(S/T)(A/I/L/V/M/T) (14). Furthermore, 80 glycoproteins were predicted in *T. forsythia* OMVs alone (10). This study supports that hundreds of proteins may indeed be O-glycosylated within *Bacteroidetes* species. Our data extend the O-glycosylation motif to (D)(S/T)(A/I/L/V/M/T/S/C/G/F), which significantly increases the number of possible glycosylation sites, at least in *T. forsythia.* It was noted previously that all amino acid side chains in the third position of the published motif contain a free methyl group that may contribute to the selectivity (12). However, none of the new amino acids (S/C/G/F) have this methyl group, indicating that the selectivity is much broader. For TfsB, the new motif predicts four additional sites (DSF, DTF, and DSS 2×), with three of these being identified. For TfsA, the new motif predicts two additional sites (DSS and DTS), with just DSS being identified as glycosylated (see Table S1 in the supplemental material). The new motifs are also commonly present in protein sequences of other *Bacteroidetes*, including Porphyromonas gingivalis; however, the occurrence of glycosylation at these motifs needs to be demonstrated experimentally.

In the earlier B. fragilis study, it was suggested that O-glycosylation provided a stabilizing function to the glycoproteins, which was supported by the apparent instability of a protein mutated to prevent its glycosylation (12). While *T. forsythia* may also use this stabilizing function, it apparently also utilizes the O-glycosylation system for virulence, as *T. forsythia* mutants producing truncated glycans display differential behaviors with regard to biofilm formation and the elicited immune response (25). These modulations should be expected, since the S-layer proteins and other T9SS cargo proteins that decorate the cell surface and the surface of released outer membrane vesicles are so heavily glycosylated (Table S2), changing their surface properties and virulence.

The presence of shorter glycans in this study is interesting, either lacking the terminal three sugars or just the Pse residue (Fig. 2B). The data suggest that the corresponding activities of the GtfI and GtfS glycosyltransferases (16) were incomplete. Besides shorter variants, a single novel glycoform was identified in this study at Δmass 1,878 corresponding to an additional deoxyhexose residue (Fig. 4B). The location of this sugar in the structure is unclear. It could be bonded to the 4Me-Gal residue as suggested in the earlier report (11), but this suggestion was unable to be confirmed (16). Alternatively, perhaps the Dig and Fuc residues bonded to the reducing end Gal are interchangeable to some extent such that Gal-(Fuc)_2_ and Gal-(Dig)_2_ configurations are produced at low frequency. This could be due to enzyme promiscuity.

Fucose was reported to be transferred to the reducing end Gal by the enzyme GtfE (16). In the *gtfE* mutant, the presence of only a residual pentasaccharide was reported, suggesting that further elongation of the glycan is dependent on this step. This conclusion, however, appears to be inconsistent with the data in both the previous studies (11, 16) and ours, which show the presence of abundant glycoforms lacking this Fuc residue, which in this study is represented by the Δmass 1,586 and 1,716 forms together with their shorter variants (Fig. 2). Possible explanations for this discrepancy include the removal of the Fuc residue after synthesis of the glycan is complete or that GtfE instead transfers the internal Fuc residue, which is necessary for further elongation of the glycan.

It was interesting to observe an apparent specificity of some glycoforms preferring certain protein sites over others (Fig. S4). This specificity appeared to relate to the presence or absence of Fuc, whereas the presence or absence of Dig had no discernible effect. Potentially, the specificity may arise due to an interaction between the protein substrate and the fucose or otherwise steric hindrance produced by the presence of the fucose.

The use of TFMS to partially cleave the glycans proved to be a powerful technique in this study. TFMS rapidly cleaved the Fuc-GlcA, Xyl-GlcA, Fuc-Gal, and Dig-Gal bonds, while the GlcA-Gal bond was relatively resistant to cleavage. This resulted in the many glycoforms shown in Fig. 2B to be converted mostly to the same residual glycan comprised of GlcA-Gal with a Δmass of 338 Da. Now having a small and abundant modification, the glycopeptides were much more readily identified. Furthermore, these less complex glycopeptides were more amenable to ETD analysis, enabling glycosylation sites to be directly confirmed (Fig. 6). In our previous study, TFMS was also shown to have utility in the analysis of the linking sugars of the T9SS (24). In that study, the reducing ends of the sugars were distal to the protein, and cleavage with TFMS in the presence of an arene resulted in arylation of the cleaved sugar at the reducing end. In the present study, the reducing ends of the sugars are proximal to the protein such that TFMS cleavage caused no modification of the protein-linked glycan. Taken together, partial deglycosylation with TFMS is a useful tool in both situations and should be considered more widely in the glycoproteomics field.

## MATERIALS AND METHODS

### Growth and fractionation of *T. forsythia*.

*T. forsythia* ATCC 43037 cells were grown anaerobically in broth, harvested, and fractionated by the use of a sonication probe and centrifugation to produce a membrane-enriched fraction as previously described (9).

### Partial deglycosylation.

Portions of the membrane fraction were resuspended in 50% acetonitrile–0.1% aqueous trifluoroacetic acid (TFA). The samples were transferred to glass vials, freeze-dried thoroughly, and deglycosylated with trifluoromethanesulfonic acid (TFMS) as previously described (24). TFMS is highly volatile and corrosive and must be used with care in a fume hood. Briefly, samples were placed in an ethanol-dry ice bath for the slow addition of a solution comprising 90% TFMS and 10% anhydrous toluene. The samples were then transferred to −20°C and the deglycosylation reaction allowed to proceed for 25 min. The reaction mixture was slowly neutralized on an ethanol-dry ice bath with three volumes of pyridine-methanol-water at a ratio of 3:1:1. Ammonium bicarbonate (50 mM, 8 vol) was then added. Proteins were recovered by precipitation with 13% trichloroacetic acid and washing with ice-cold acetone.

### SDS-PAGE and in-gel digestion.

Untreated and deglycosylated membrane protein samples were separated by SDS-PAGE and fractionated into 12 or 16 bands as shown in Fig. S1 in the supplemental material. The bands were in-gel digested with trypsin as previously described (9) and extracted once with 0.1% aqueous TFA and once with 30% acetonitrile–0.1% aqueous TFA, both for 15 min in an ultrasonication bath. Extracts were combined, evaporated in a vacuum centrifuge and dissolved in 2% acetonitrile–0.1% aqueous TFA ready for MS analysis.

### Mass spectrometry.

LC-MS/MS experiments were conducted on a Dionex Ultimate 3000 UHPLC interfaced with an Orbitrap Fusion Lumos Tribrid mass spectrometer (Thermo Fisher Scientific) as previously described (26), with the following modifications. For the analysis of intact glycopeptides, peptides were eluted over a 60-min gradient from approximately 2 to 40% acetonitrile. A stepped FAIMS method was employed, alternating between −25 V and −45 V. Specific glycan fragment ions (199.071, 345.129, and 362.156) were used to trigger the additional CID, EThcD, and HCD scans with previously described scanning parameters (26).

For the analysis of acid-cleaved glycopeptides, peptides were eluted over a 22-min gradient from approximately 2 to 20% acetonitrile and then up to 32% acetonitrile in a further 2 min. The FAIMS setting was constant at −45 V, and only HCD spectra were collected. For the collection of ETD and CID spectra, selected samples were analyzed again using the same LC-MS/MS method, except that previously identified glycopeptides were preferred by the use of a mass inclusion list. HCD, ETD, EThcD, and CID scans were performed on all.

### Peptide identification.

Proteins and peptides were identified by searching against the *T. forsythia* ATCC 43037 sequence database (GenBank accession no. JUET01000058.1) (27) with corrections as previously described (9). All searches were conducted using trypsin with up to two missed cleavages allowed. Peptide mass tolerance was set to 10 ppm and fragment mass tolerance to 0.04 Da, with a fixed modification of carbamidomethyl (C). Initially, the raw MS data was searched with Byonic v3.9.6 (Protein Metrics Inc.) using the wildcard setting, initially set to 100 to 2,000 Da and later extended to 2,500 Da (28). After identification of most of the glycan additions, the data were searched again using specific modifications as follows. Common modifications were oxidation (M) and 1,586.54 (S,T). Rare modifications were, all on S,T, 792.25, 922.25, 938.31, 1,068.37, 1,225.39, 1,355.45, 1,716.60, 1,732.60, 1,862.65, 1,876.66, and 1,878.65. A maximum of 5 common and 2 rare modifications were allowed. For the identification of acid-cleaved glycopeptides, Mascot v2.6.2 (Matrix Science, UK) was used in error-tolerant mode, with oxidation (M) and Hex-HexA (ST) (monoisotopic mass = 338.0849) set as variable modifications. Only modification fragments specific to the *T. forsythia* glycan were added to the list of modifications used for the error-tolerant search. A glycopeptide was considered identified and included in Table S1 if it contained the sequence of the glycosylation motif and was identified by at least one glycopeptide with a Byonic score greater than 200 and −log_10_ (*P*) > 1 or a Mascot score greater than 30. The false discovery rate (FDR) of identifying individual glycopeptides with these thresholds was 0.25% for Byonic. Since decoy mode is not compatible with error-tolerant mode, the FDR for the Mascot searches was calculated for the most abundant modification (Hex-HexA), which was used as a variable modification. The FDR was insignificant, as no peptides with the Hex-HexA modification were identified in the decoy database with a score of >30. Where multiple glycopeptides of different sequence or glycan addition were identified for the same site, lower Byonic or Mascot scores were permitted. MS/MS spectral matches relying on a Byonic score between 200 and 300 were manually checked for the presence of signature low mass ions corresponding to the amino sugars, strong matches to b or y ion series, and a strong peak matching to the full-length peptide (since the sugars were preferably cleaved off by HCD fragmentation).

### Protein localization.

The subcellular localization of proteins was predicted based on published findings and methods (9). Outer membrane β-barrel predictive scores (TMBB) were obtained using the Freeman Wimley analysis tool obtained from the Wimley Laboratory (http://beta-barrel.tulane.edu/). The program was used without considering the SignalP predictions (29). The Pfam data were obtained by performing a batch sequence search using all *T. forsythia* protein sequences against the Pfam 27.0 database at pfam.xfam.org. Inner membrane proteins (IMPs) were predicted by homology and with the help of the TMHMM Server v2.0 (www.cbs.dtu.dk/services/TMHMM-2.0/), where 4 predicted transmembrane helices was regarded as strongly predictive of IM location.

### Data availability.

The mass spectrometry proteomics data have been deposited in the ProteomeXchange Consortium via the PRIDE (1) partner repository with the data set identifiers PXD026989 and 10.6019/PXD026989.
