# Supplementary material for: Characterization of the O-Glycoproteome of Tannerella forsythia
Source: mSphere. 2021 Sep 15;6(5):e00649-21. doi: 10.1128/mSphere.00649-21 (PMC8550257; doi:10.1128/mSphere.00649-21)
Supplement: FIG S1 [file msphere.00649-21-sf001.docx]

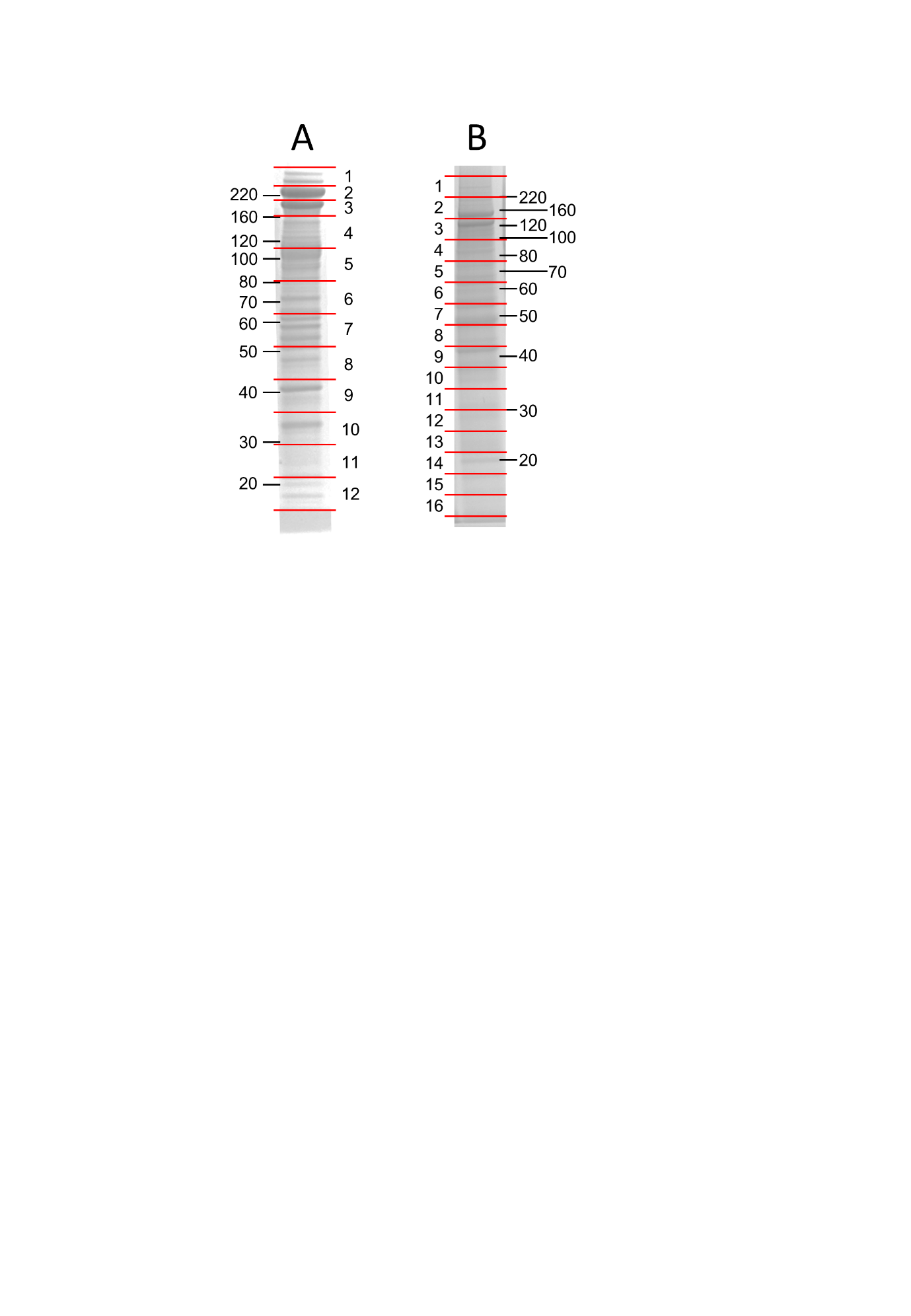


**Fig S1. SDS-PAGE of membrane-enriched fractions.** A. Untreated sample for analysis of intact glycans. B. Partially deglycosylated sample for analysis of truncated glycans. The gel lanes were excised into 12 or 16 sections as indicated and digested in-gel with trypsin prior to LC-MS/MS analyses.
