# Supplementary material for: Characterization of the O-Glycoproteome of Tannerella forsythia
Source: mSphere. 2021 Sep 15;6(5):e00649-21. doi: 10.1128/mSphere.00649-21 (PMC8550257; doi:10.1128/mSphere.00649-21)
Supplement: FIG S2 [file msphere.00649-21-sf002.docx]

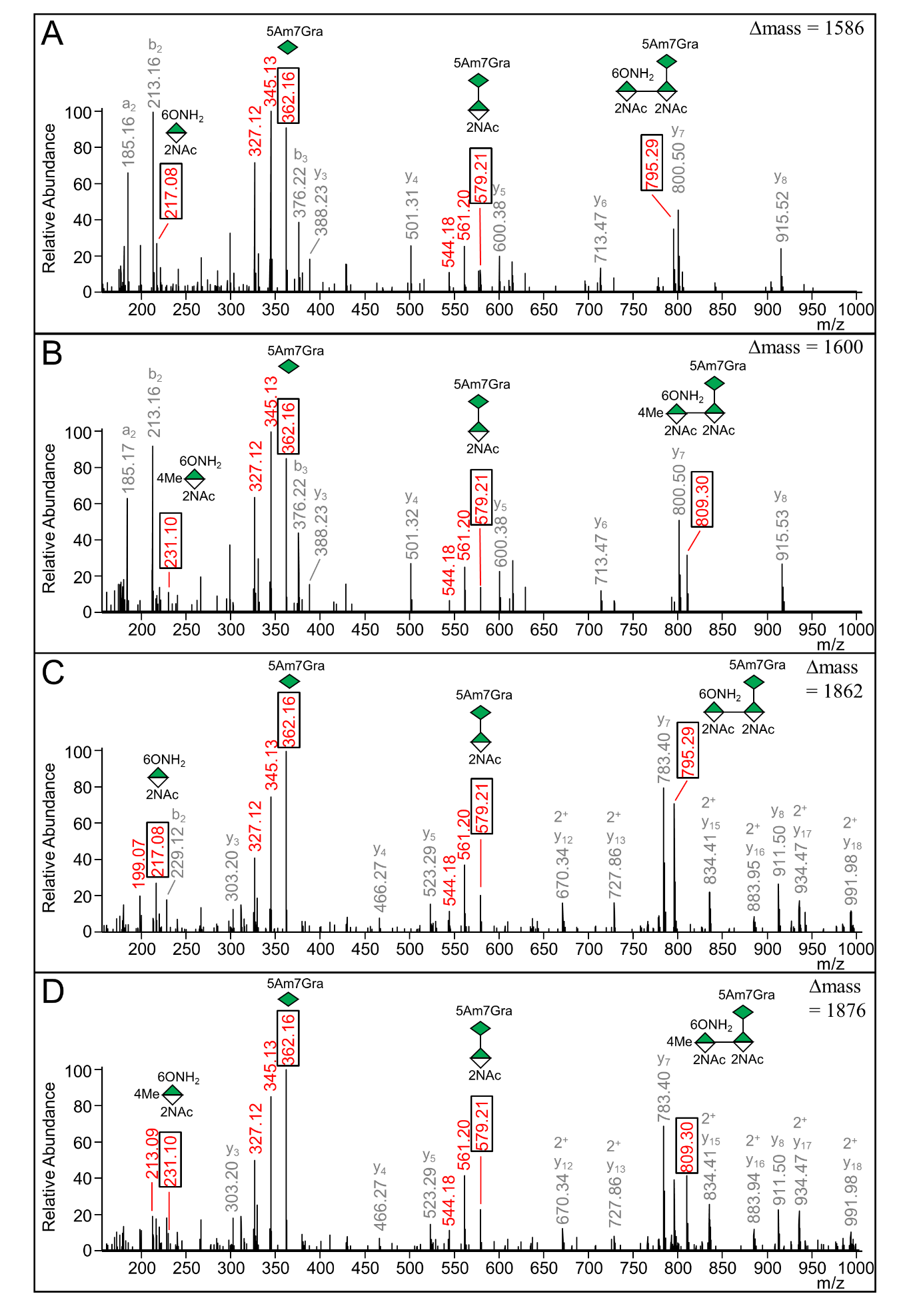


**Fig S2. Low mass region of HCD spectra confirm methylation of N-acetyl** **mannosaminuronamide.** A. MS/MS (HCD) spectrum of m/z = 1118.497 (3^+^) matching to the peptide sequence LVYYPSEDSLVINVR (+1586 Da) from Tanf_03370. B. MS/MS (HCD) spectrum of m/z = 1123.155 (3^+^) matching to the peptide sequence LVYYPSEDSLVINVR (+1600 Da) from Tanf_03370. C. MS/MS (HCD) spectrum of m/z = 990.432 (4^+^) matching to the peptide sequence IDTVNVDAGHYKPYGYGVK (+1862 Da) from Tanf_03375. D. MS/MS (HCD) spectrum of m/z = 993.931 (4^+^) matching to the peptide sequence IDTVNVDAGHYKPYGYGVK (+1876 Da) from Tanf_03375. The boxed glycan fragment ions are singly charged and correspond to the indicated structures. Non-boxed ions shown in red represent losses of water or ammonia from the boxed ions. Ions corresponding to peptide fragments are labeled in grey.
