# Supplementary material for: Characterization of the O-Glycoproteome of Tannerella forsythia
Source: mSphere. 2021 Sep 15;6(5):e00649-21. doi: 10.1128/mSphere.00649-21 (PMC8550257; doi:10.1128/mSphere.00649-21)
Supplement: FIG S3 [file msphere.00649-21-sf003.pdf]

# **Figure S3**: Additional ETD and HCD spectra showing site localization of glycans

This figure contains 30 MS/MS spectra labelled A – AD that show localization (to varying confidence) to the indicated residue. The figures correspond to data shown in Table S5.

Note: ions labelled with “~” such as “~y3” have had the glycan cleaved and therefore are not helpful for localization.

A

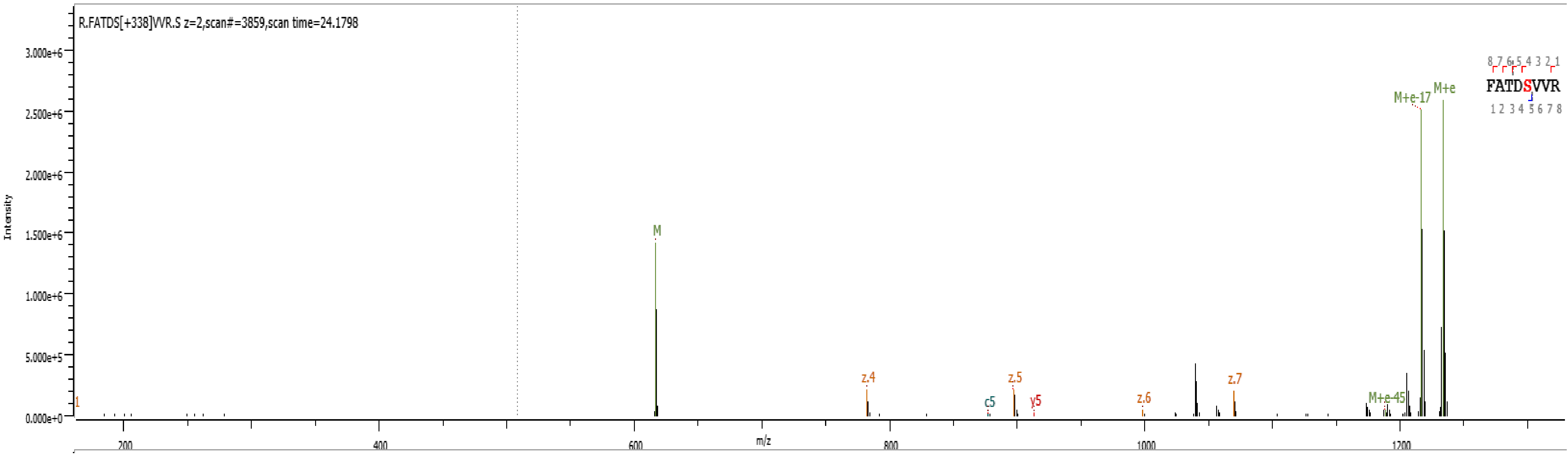

R.FATDS[+338]VVR.S z=2,scan#=3859,scan time=24.1798
Intensity
m/z
M
M+e-17
M+e
M+e-45
z.4
z.5
z.6
z.7
c5
y5
FATDSVVR

**B**

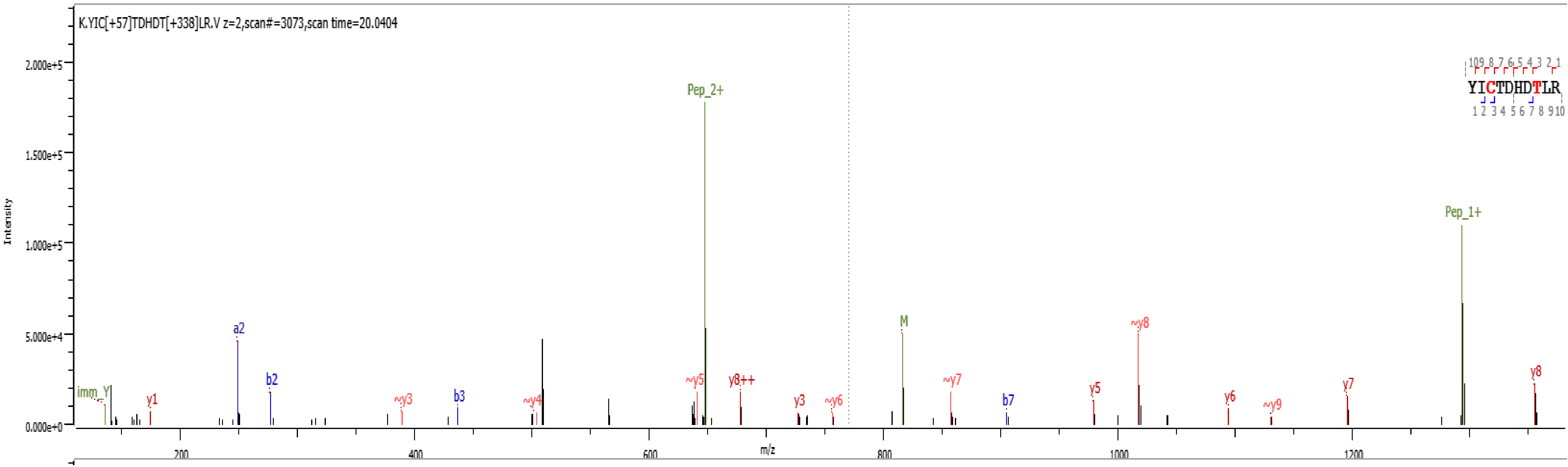
K.YIC[+57]TDHDT[+338]LR.V z=2,scan#=3073,scan time=20.0404
Intensity
m/z
Pep_2+
Pep_1+
M
a2
b2
b3
b7
imm_Y
y1
~y3
~y4
~y5
y8++
y3
~y6
~y7
y5
~y8
y6
~y9
y7
y8
YICTDHDTLR

C

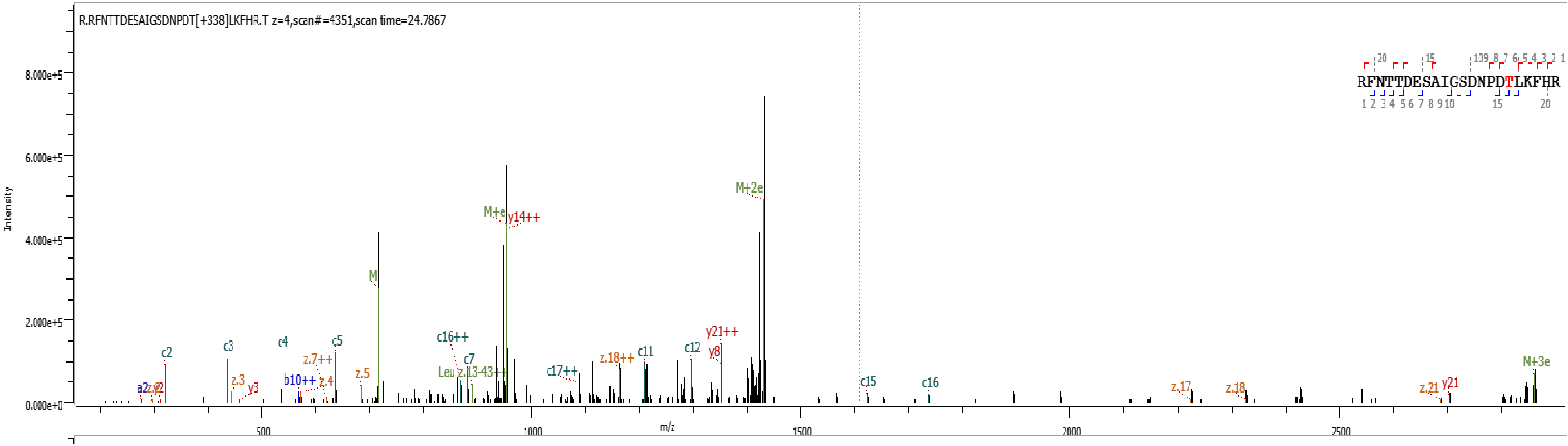
R.RFNTTDESAIGSDNPDT[+338]LKFHR.T z=4,scan#=4351,scan time=24.7867
RFNTTDESAIGSDNPDTLKFHR
Intensity
m/z
M+2e
M+e
y14++
M
c16++
c7
y21++
y8
z.18++
c11
c12
c17++
c15
c16
z.17
z.18
z.21
y21
M+3e
c2
c3
c4
c5
z.7++
z.5
b10++
z.4
z.3
y3
a2
Leu

# D

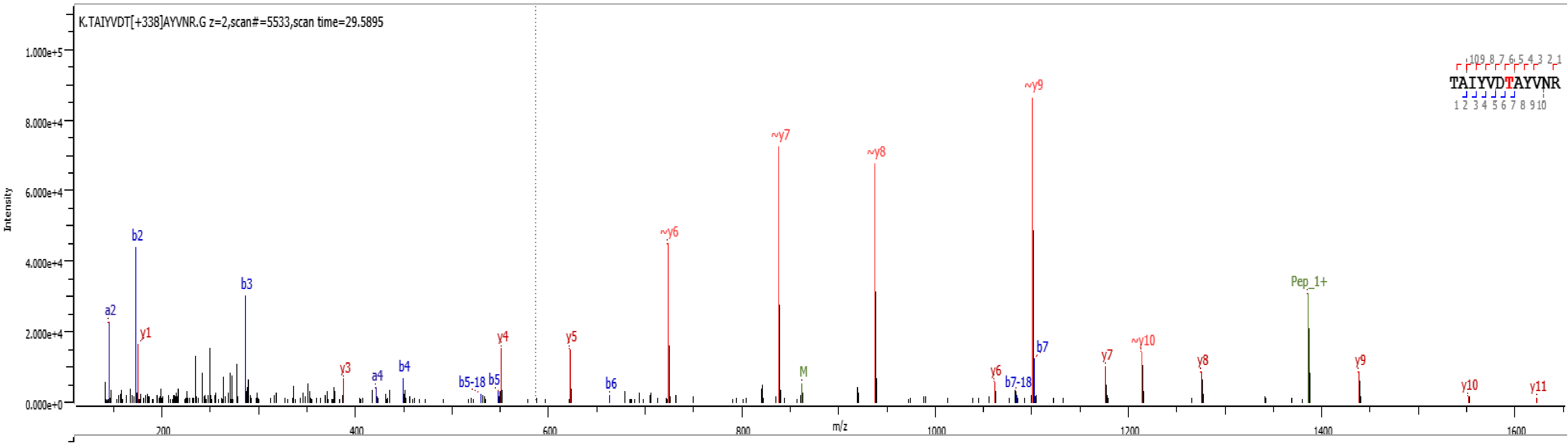
K.TAIYVDT[+338]AYVNR.G z=2,scan#=5533,scan time=29.5895
Intensity
1.000e+5
8.000e+4
6.000e+4
4.000e+4
2.000e+4
0.000e+0
m/z
200
400
600
800
1000
1200
1400
1600
a2
b2
y1
b3
y3
a4
b4
b5-18
b5
y4
y5
b6
~y6
~y7
M
~y8
y6
b7-18
~y9
b7
y7
~y10
y8
Pep_1+
y9
y10
y11
TAIYVDTAYVNR

E

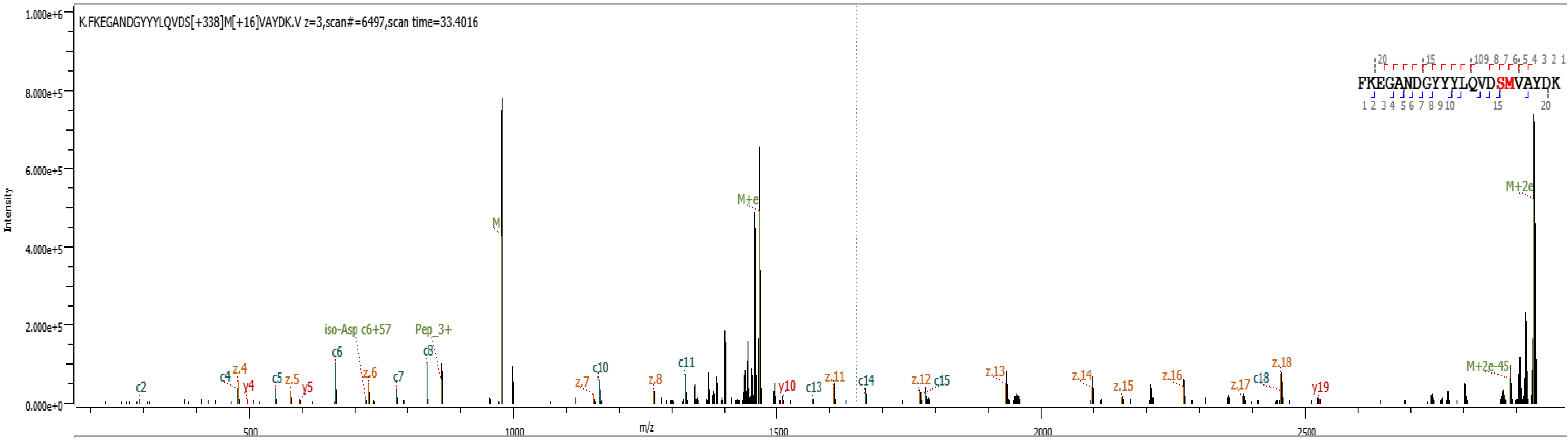
K.FKEGANDGYYYLQVDS[+338]M[+16]VAYDK.V z=3,scan#=6497,scan time=33.4016
FKEGANDGYYYLQVDSMVAYDK
Intensity
m/z
1.000e+6
8.000e+5
6.000e+5
4.000e+5
2.000e+5
0.000e+0
500
1000
1500
2000
2500
M
M+e
M+2e
M+2e-45
iso-Asp c6+57
Pep_3+
c2
c4
z.4
y4
c5
z.5
y5
c6
z.6
c7
c8
z.7
c10
z.8
c11
y10
c13
z.11
c14
z.12
c15
z.13
z.14
z.15
z.16
z.17
c18
z.18
y19

F

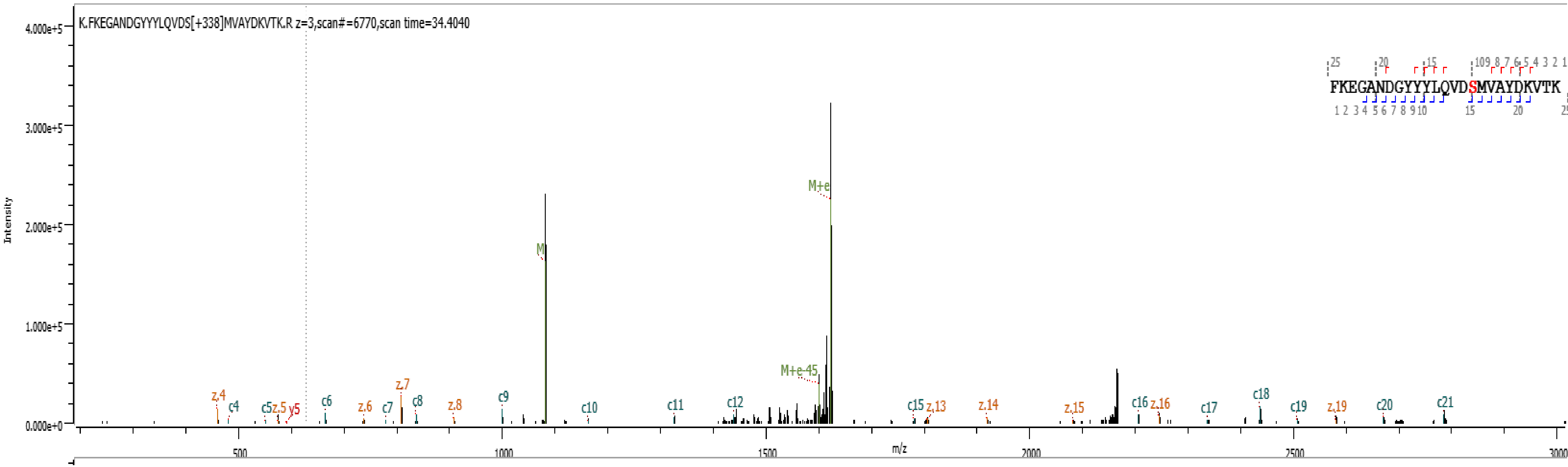

K.FKEGANDGYYYLQVDS[+338]MVAYDKVTK.R z=3,scan#=6770,scan time=34.4040
FKEGANDGYYYLQVDSMVAYDKVTK
Intensity
m/z
4.000e+5
3.000e+5
2.000e+5
1.000e+5
0.000e+0
500
1000
1500
2000
2500
3000
M
M+e
M+e-45
z.4
c4
c5
z.5
y5
c6
z.6
c7
z.7
c8
z.8
c9
c10
c11
c12
c15
z.13
z.14
z.15
c16
z.16
c17
c18
c19
z.19
c20
c21

G

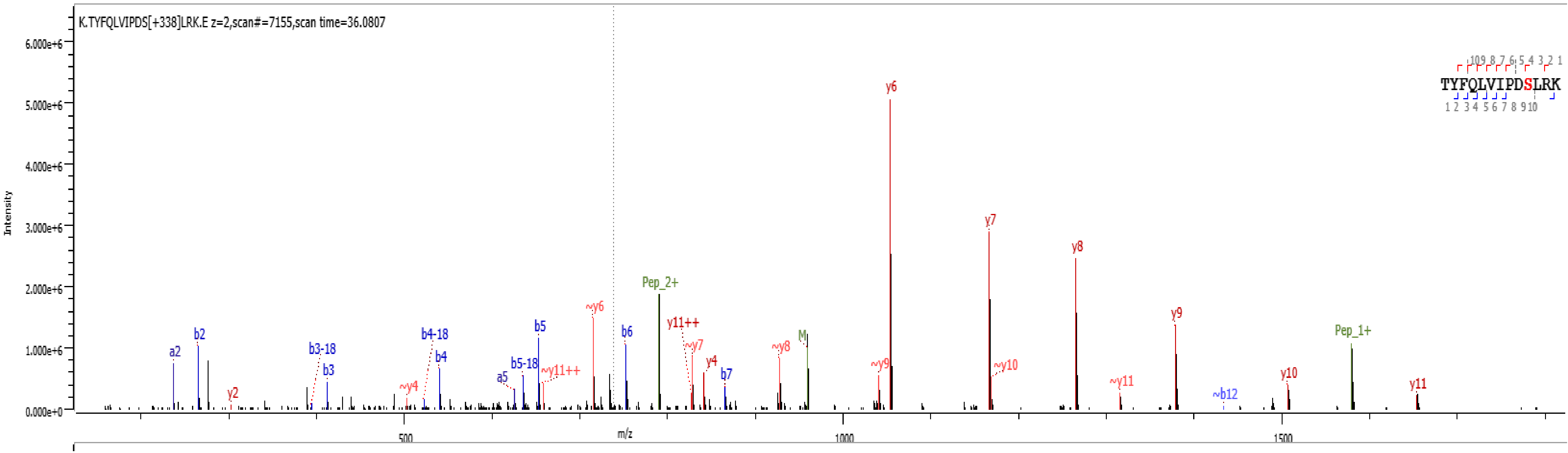
K.TYFQLVIPDS[+338]LRK.E z=2,scan#=7155,scan time=36.0807
Intensity
m/z
TYFQLVIPDSLRK
y6
y7
y8
y9
y10
y11
Pep_2+
Pep_1+
y11++
b2
b5
b6
b7
a2
b3-18
b4-18
b4
b3
b5-18
a5
~y6
~y11++
~y7
y4
~y8
~y9
~y10
~y11
~b12
y2
~y4
M

H

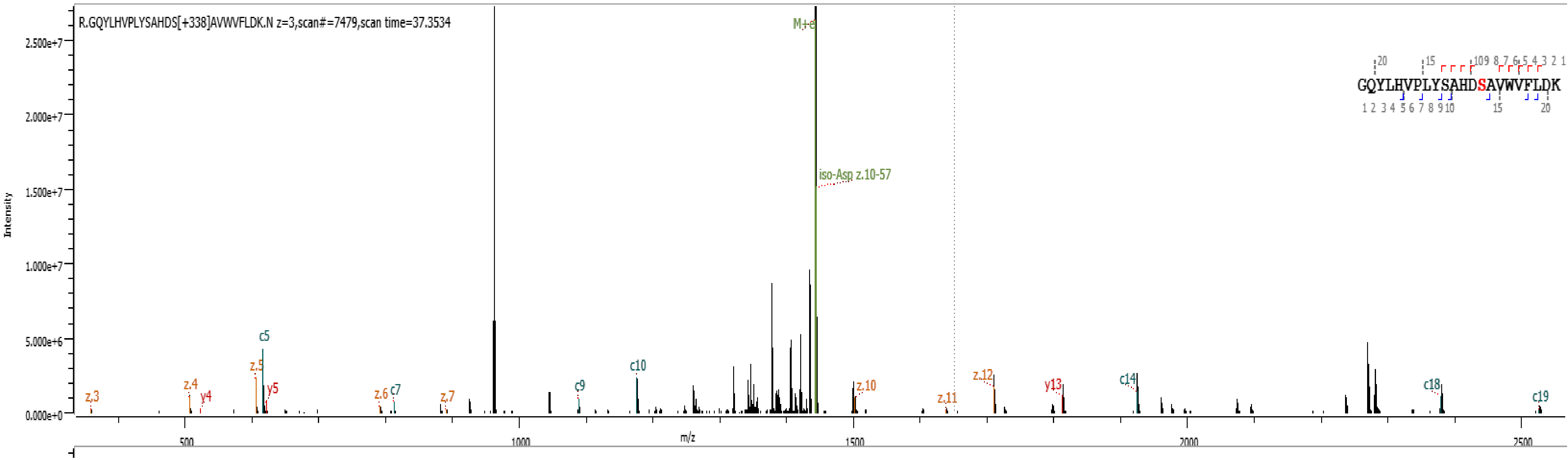

R.GQYLHVPLYSAHDS[+338]AVWVFLDK.N z=3,scan#=7479,scan time=37.3534
M+e
iso-Asp z.10-57
GQYLHVPLYSAHDSAVWVFLDK
Intensity
m/z

I

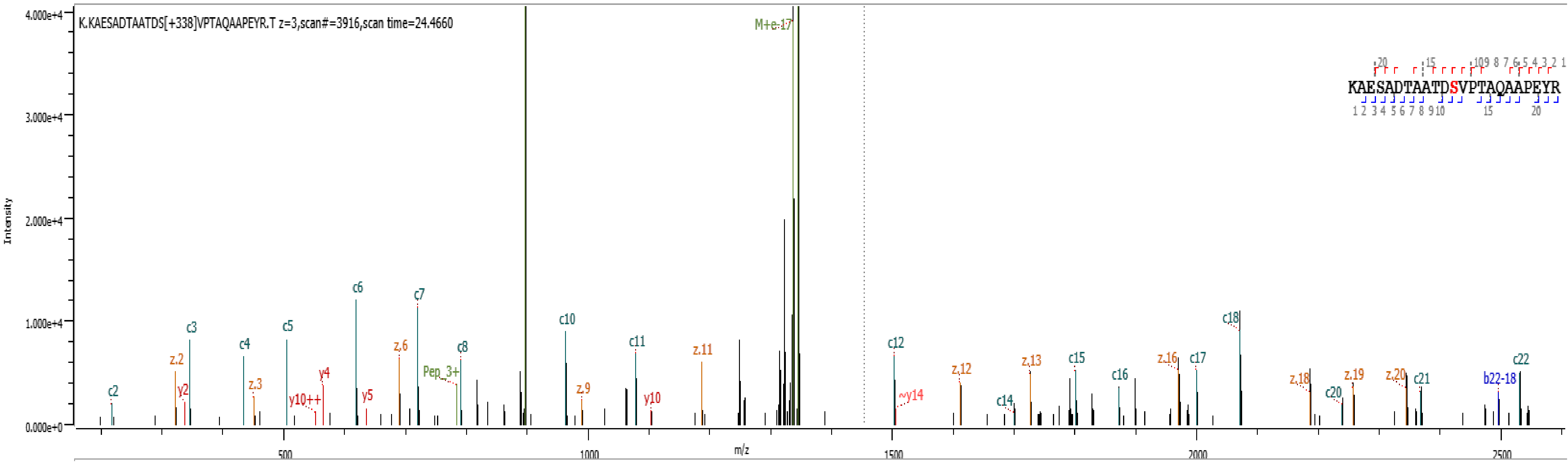
K.KAESADTAATDS[+338]VPTAQAAPEYR.T z=3,scan#=3916,scan time=24.4660
Intensity
m/z
M+e-17
Pep_3+
KAESADTAATDSVPTAQAAPEYR
c2
c3
c4
c5
c6
c7
c8
c10
c11
c12
c14
c15
c16
c17
c18
c20
c21
c22
z.2
z.3
z.6
z.9
z.11
z.12
z.13
z.16
z.18
z.19
z.20
y2
y4
y5
y10++
y10
~y14
b22-18

J

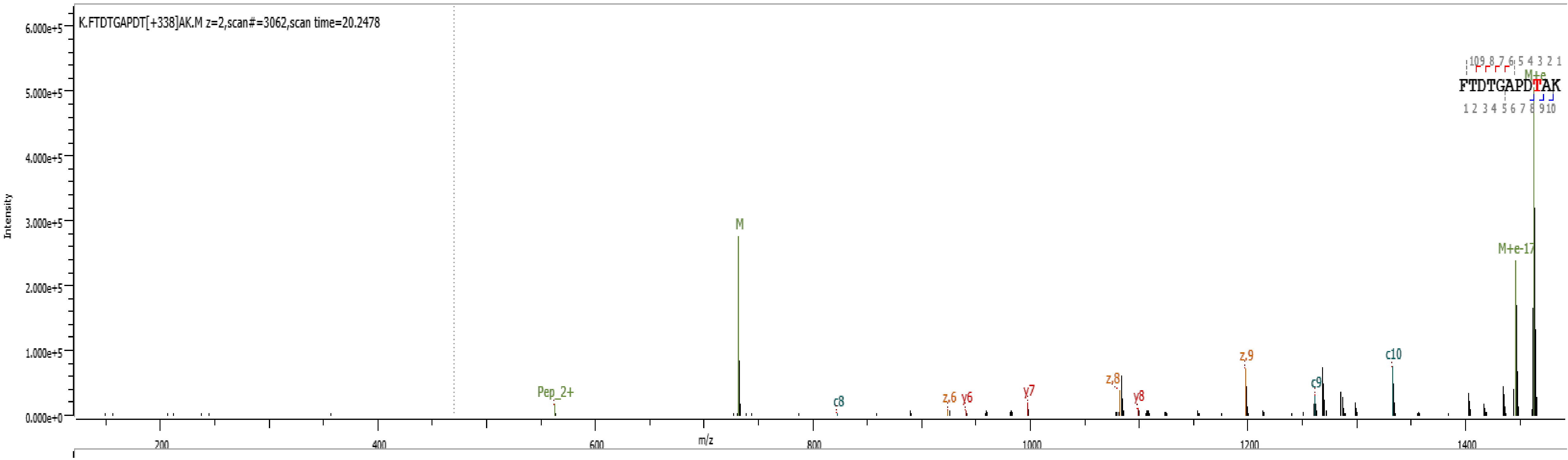

K.FTDTGAPDT[+338]AK.M z=2,scan#=3062,scan time=20.2478
Intensity
6.000e+5
5.000e+5
4.000e+5
3.000e+5
2.000e+5
1.000e+5
0.000e+0
m/z
200
400
600
800
1000
1200
1400
FTDTGAPDTAK
M+e
M
M+e-17
Pep_2+
c8
z,6
y6
y7
z,8
y8
z,9
c9
c10

K

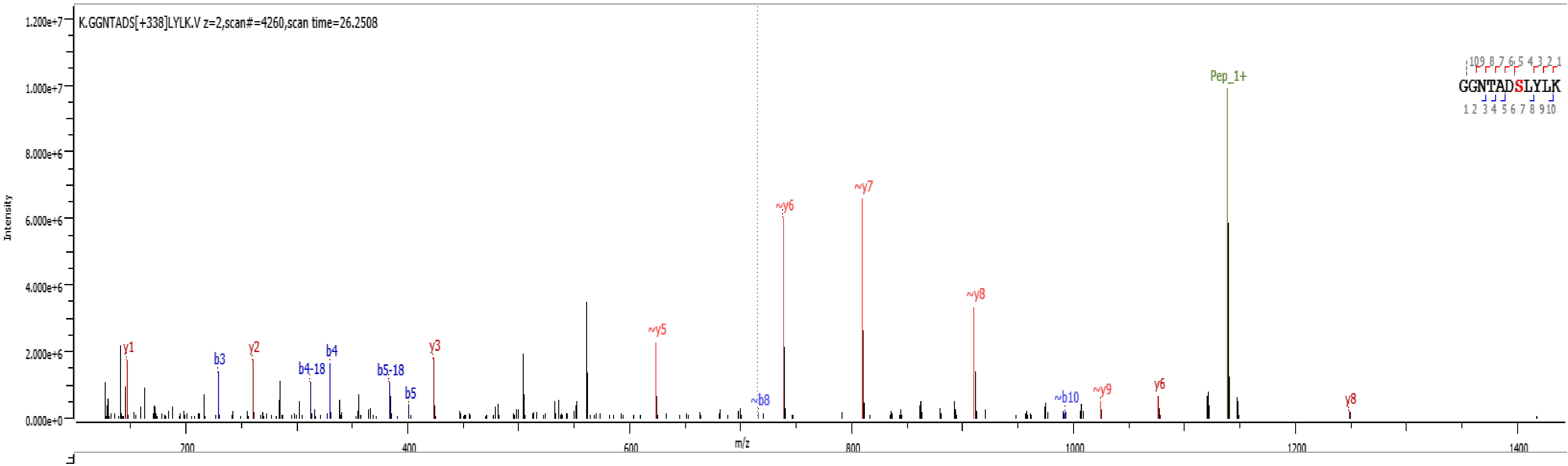
K.GGNTADS[+338]LYLK.V z=2,scan#=4260,scan time=26.2508
Intensity
m/z
Pep_1+
~y7
~y6
~y8
~y5
y1
b3
y2
b4-18
b4
b5-18
b5
y3
~b8
~b10
~y9
y6
y8
GGNTADSLYLK
1.200e+7
1.000e+7
8.000e+6
6.000e+6
4.000e+6
2.000e+6
0.000e+0
200
400
600
800
1000
1200
1400

L

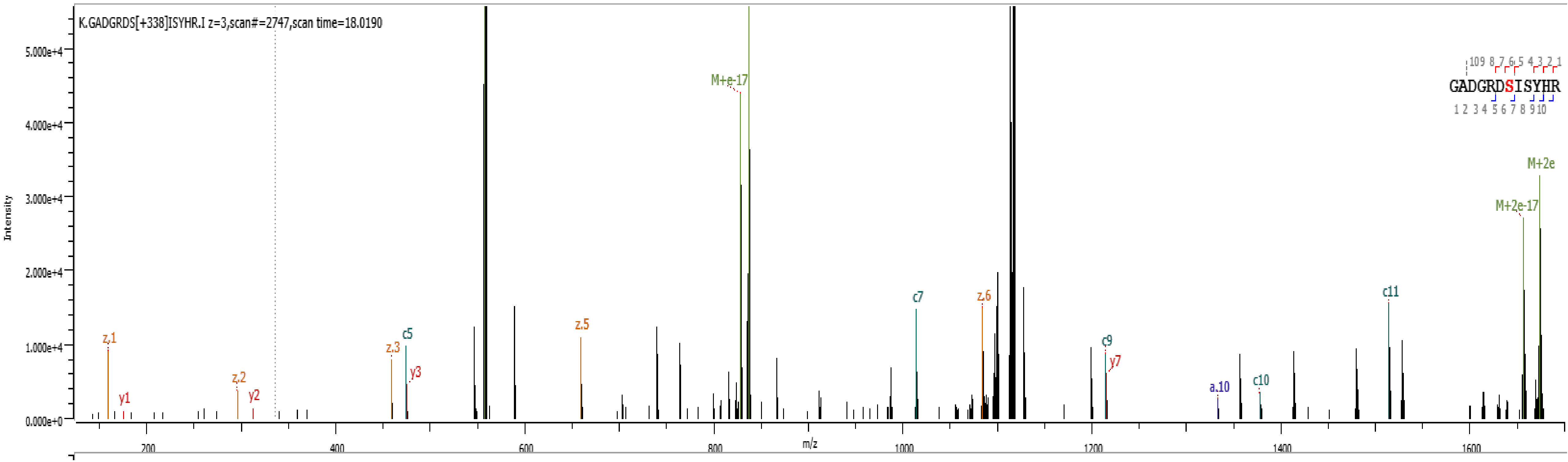
K.GADGRDS[+338]ISYHR.I z=3,scan#=2747,scan time=18.0190
Intensity
m/z
GADGRDSISYHR
M+e-17
M+2e
M+2e-17
z.1
y1
z.2
y2
z.3
c5
y3
z.5
c7
z.6
c9
y7
a.10
c10
c11

# M

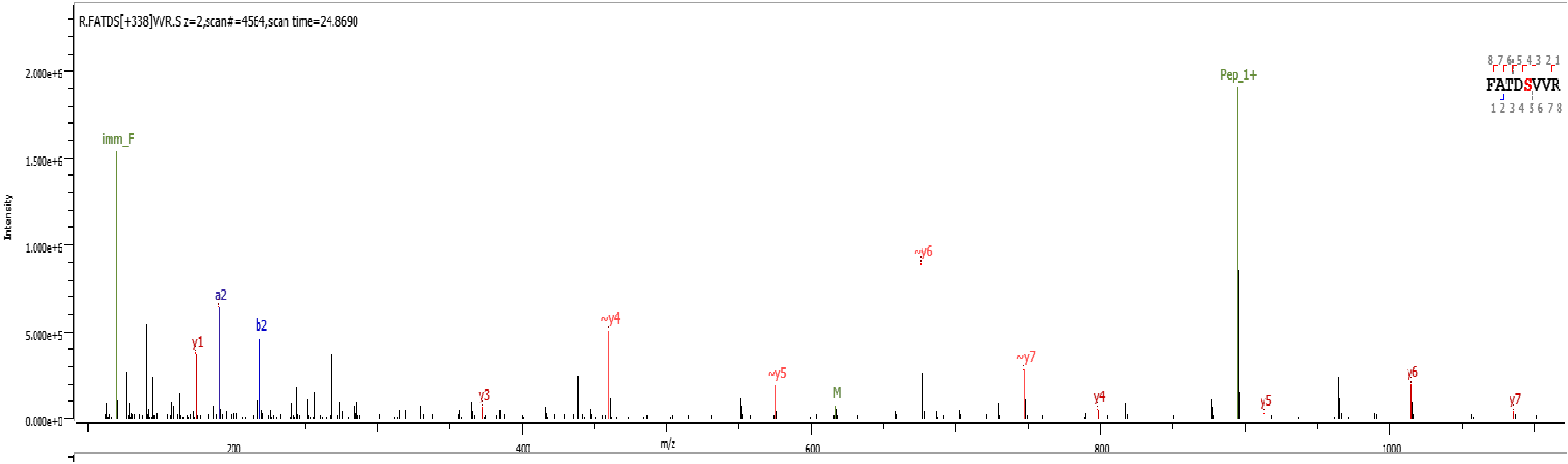

R.FATDS[+338]VVR.S z=2,scan#=4564,scan time=24.8690
Intensity
m/z
imm_F
y1
a2
b2
y3
~y4
~y5
M
~y6
~y7
y4
Pep_1+
y5
y6
y7
FATDSVVR

# N

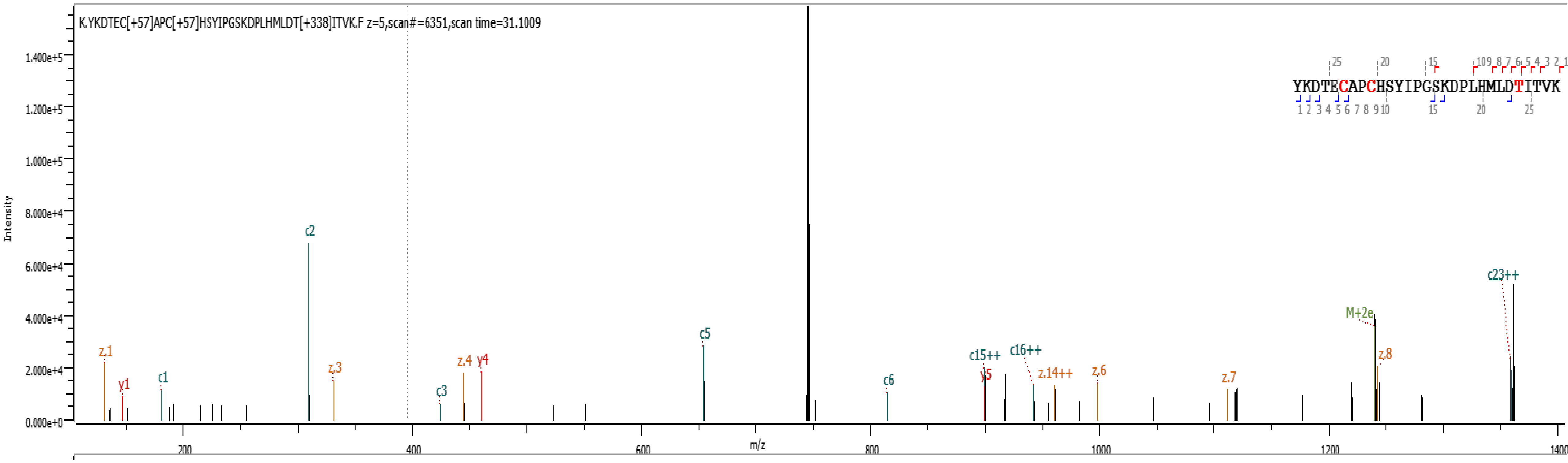
K.YKDTEC[+57]APC[+57]HSYIPGSKDPLHMLDT[+338]ITVK.F z=5,scan#=6351,scan time=31.1009
YKDTECAPCHSYIPGSKDPLHMLDTITVK
Intensity
m/z
1.400e+5
1.200e+5
1.000e+5
8.000e+4
6.000e+4
4.000e+4
2.000e+4
0.000e+0
200
400
600
800
1000
1200
1400
z.1
y1
c1
c2
z.3
c3
z.4
y4
c5
c6
c15++
y5
c16++
z.14++
z.6
z.7
M+2e
z.8
c23++

**O**

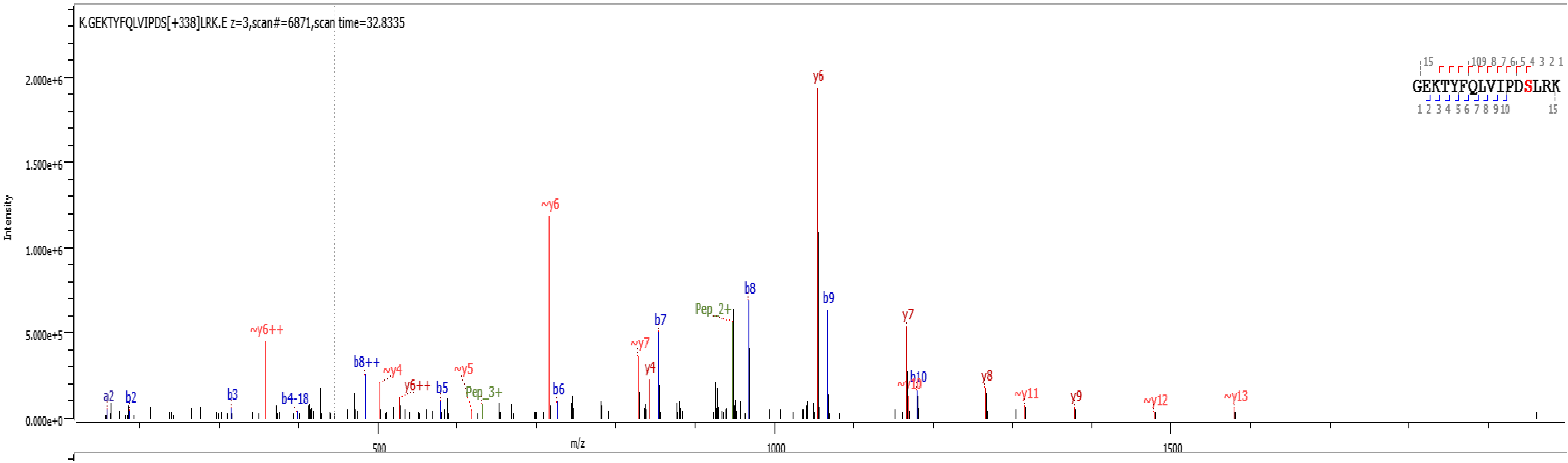

K.GEKTYFQLVIPDS[+338]LRK.E z=3,scan#=6871,scan time=32.8335
Intensity
m/z
GEKTYFQLVIPDSLRK
y6
~y6
~y6++
b8
b9
Pep_2+
b7
~y7
y4
y7
b8++
~y4
~y5
y6++
b5
Pep_3+
b6
b3
b4-18
a2
b2
b10
y8
~y11
y9
~y12
~y13

# P

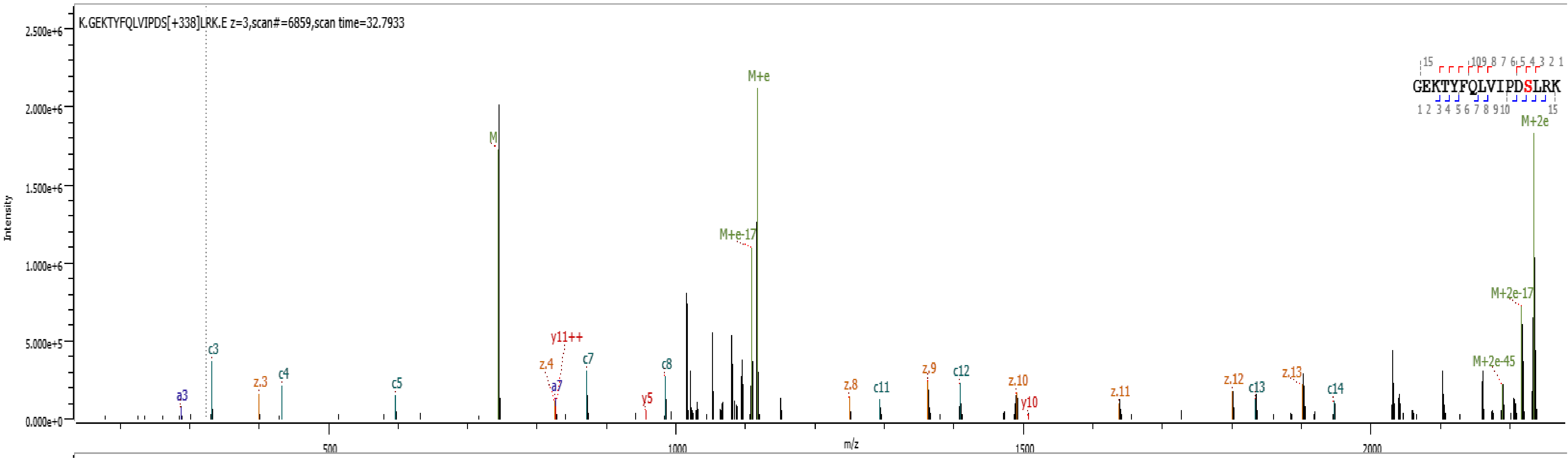

K.GEKTYFQLVIPDS[+338]LRK.E z=3,scan#=6859,scan time=32.7933
Intensity
m/z
GEKTYFQLVIPDSLRK
M+e
M
M+e-17
M+2e
M+2e-17
M+2e-45
y11++
y5
y10
a3
a7
c3
c4
c5
c7
c8
c11
c12
c13
c14
z.3
z.4
z.8
z.9
z.10
z.11
z.12
z.13

Q

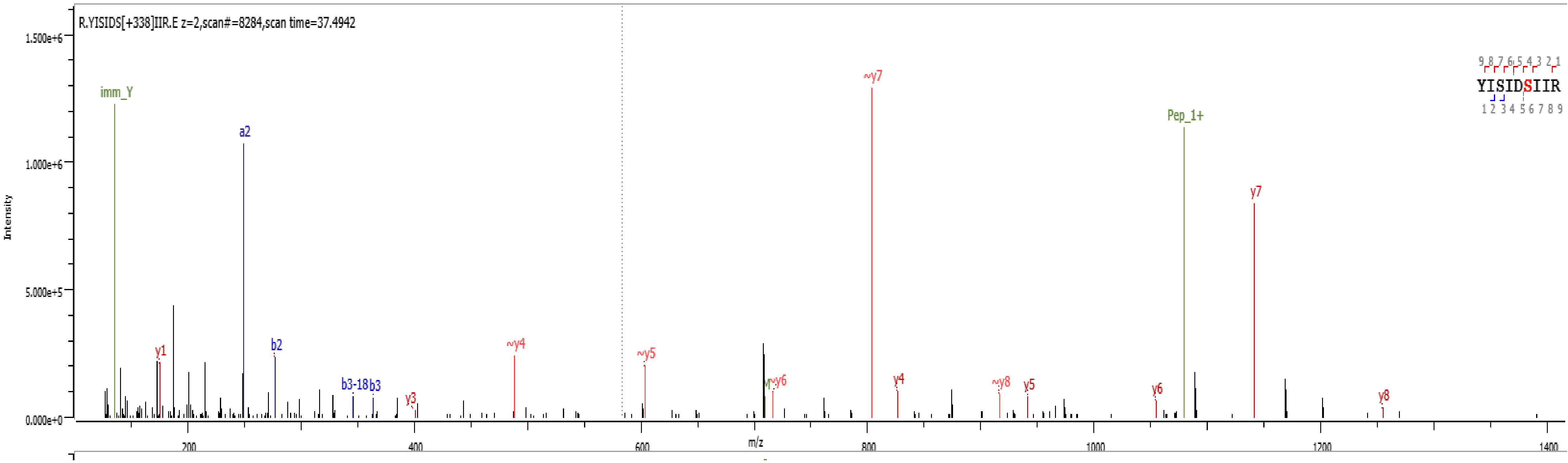
R.YISIDS[+338]IIR.E z=2,scan#=8284,scan time=37.4942
Intensity
m/z
imm_Y
a2
b2
y1
b3-18
b3
y3
~y4
~y5
~y6
~y7
y4
~y8
y5
y6
Pep_1+
y7
y8
YISIDSIIR

R

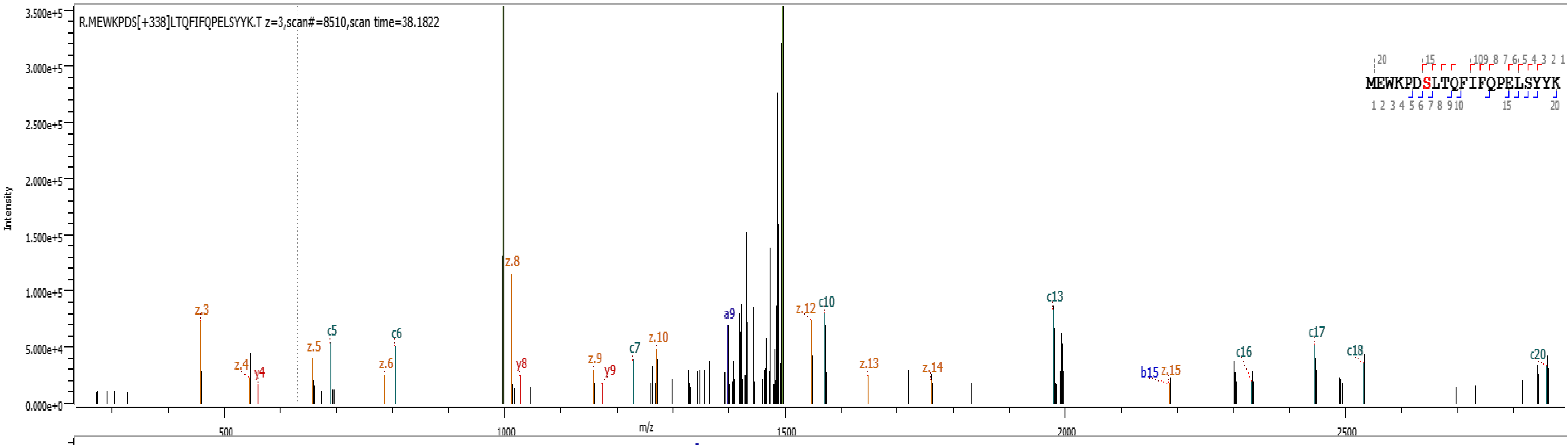
R.MEWKPDS[+338]LTQFIFQPELSYYK.T z=3,scan#=8510,scan time=38.1822
Intensity
m/z
MEWKPDSLTQFIFQPELSYYK
z.3
z.4
y4
z.5
c5
z.6
c6
z.8
y8
z.9
y9
c7
z.10
a9
z.12
c10
z.13
z.14
c13
b15
z.15
c16
c17
c18
c20

S

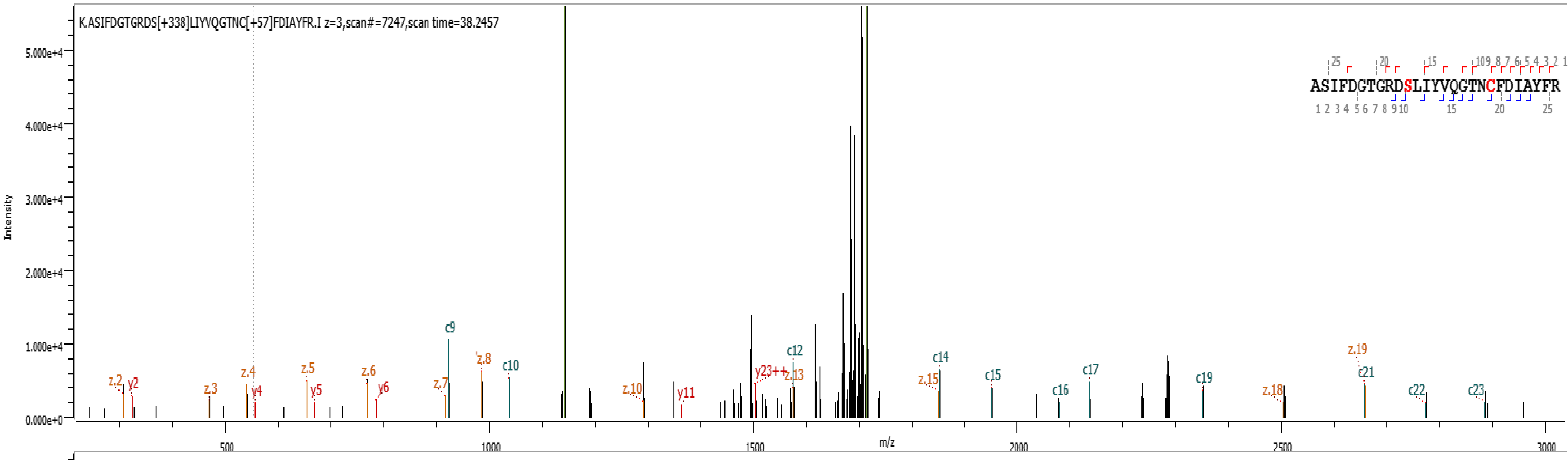

K.ASIFDGTGRDS[+338]LIYVQGTNC[+57]FDIAYFR.I z=3,scan#=7247,scan time=38.2457
Intensity
m/z
ASIFDGTGRDSLIYVQGTNCFDIAYFR

# T

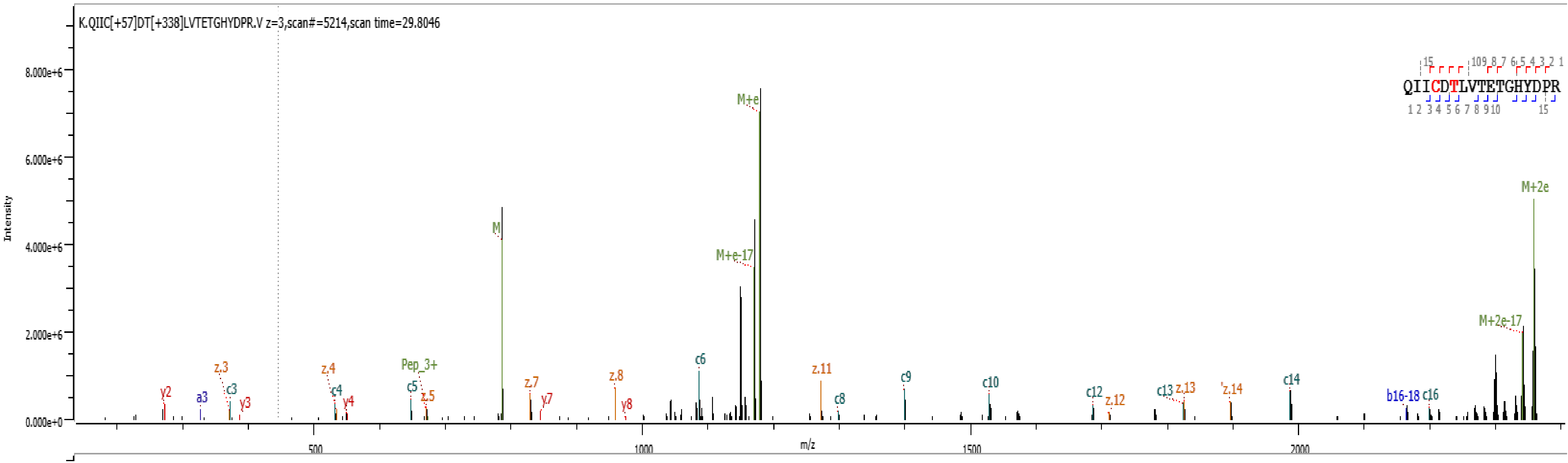

K.QIIC[+57]DT[+338]LVTETGHYDPR.V z=3,scan#=5214,scan time=29.8046
Intensity
m/z
QIICDTLVTETGHYDPR
M+e
M+e-17
M
M+2e
M+2e-17
Pep_3+
y2
a3
c3
y3
z.3
z.4
c4
y4
c5
z.5
z.7
y7
z.8
y8
c6
z.11
c8
c9
c10
c12
z.12
c13
z.13
z.14
c14
b16-18
c16

U

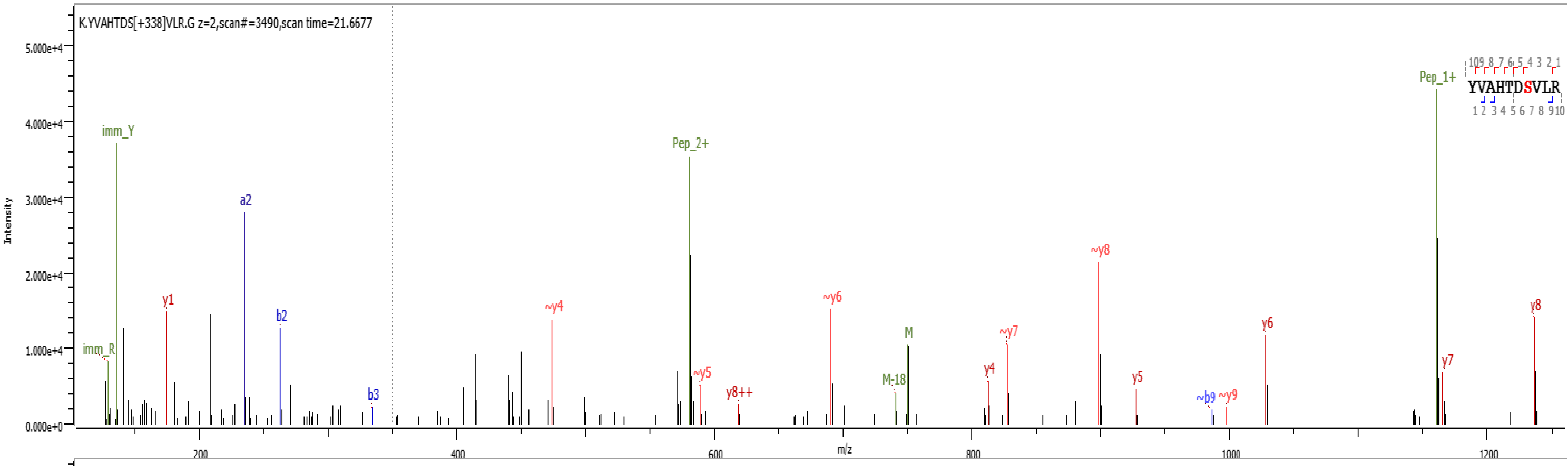
K.YVAHTDS[+338]VLR.G z=2,scan#=3490,scan time=21.6677
Intensity
m/z
YVAHTDSVLR
imm_Y
imm_R
y1
a2
b2
b3
~y4
Pep_2+
~y5
y8++
~y6
M-18
M
y4
~y7
~y8
y5
~b9
~y9
y6
Pep_1+
y7
y8

V

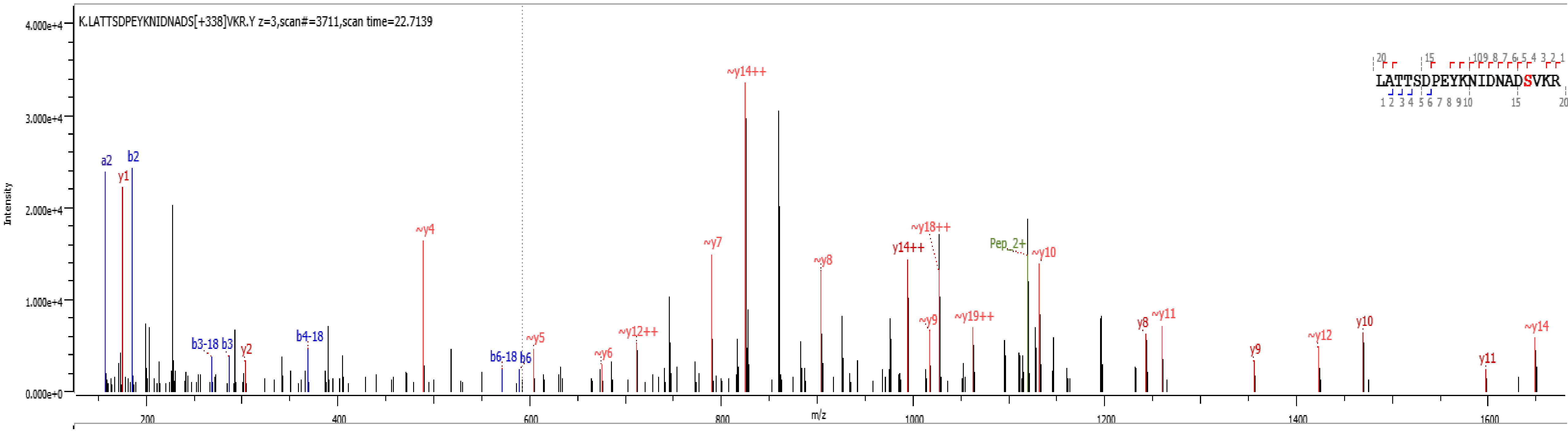
K.LATTSDPEYKNIDNADS[+338]VKR.Y z=3,scan#=3711,scan time=22.7139
Intensity
m/z
LATTSDPEYKNIDNADSVKR
a2
b2
y1
b3-18
b3
y2
b4-18
~y4
b6-18
b6
~y5
~y6
~y12++
~y7
~y14++
~y8
y14++
~y9
~y18++
~y19++
Pep_2+
~y10
y8
~y11
y9
~y12
y10
y11
~y14

W

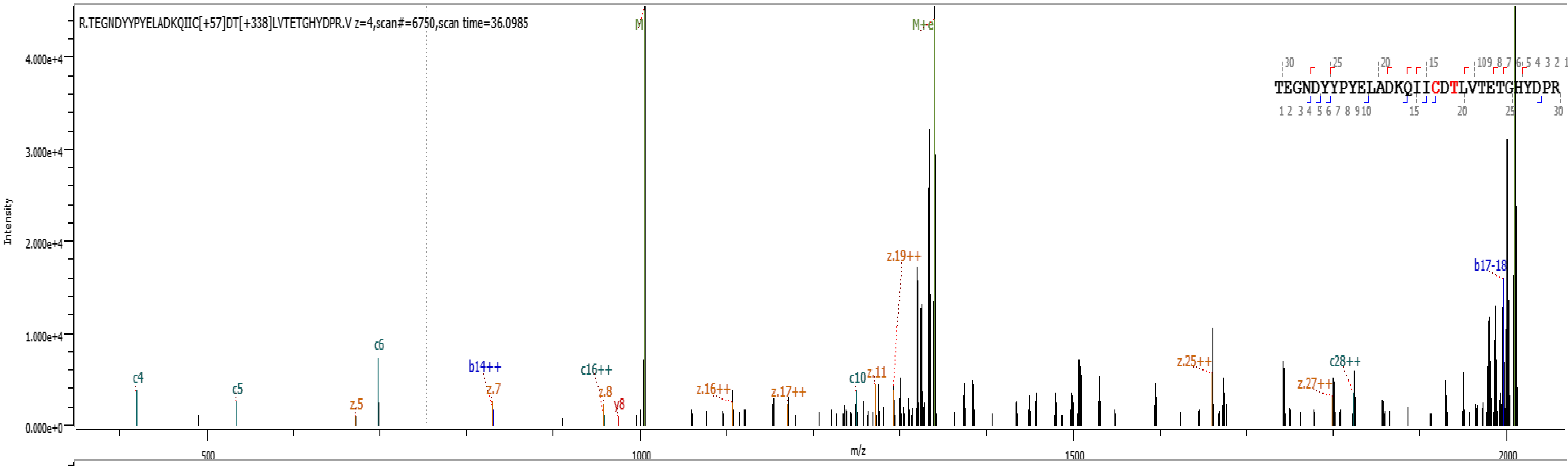
R.TEGNDYYPYELADKQIIC[+57]DT[+338]LVTETGHYDPR.V z=4,scan#=6750,scan time=36.0985
Intensity
m/z
TEGNDYYPYELADKQIICDTLVTETGHYDPR
M
M+e
c4
c5
z.5
c6
b14++
z.7
c16++
z.8
y8
z.16++
z.17++
c10
z.11
z.19++
z.25++
z.27++
c28++
b17-18
4.000e+4
3.000e+4
2.000e+4
1.000e+4
0.000e+0
500
1000
1500
2000

# X

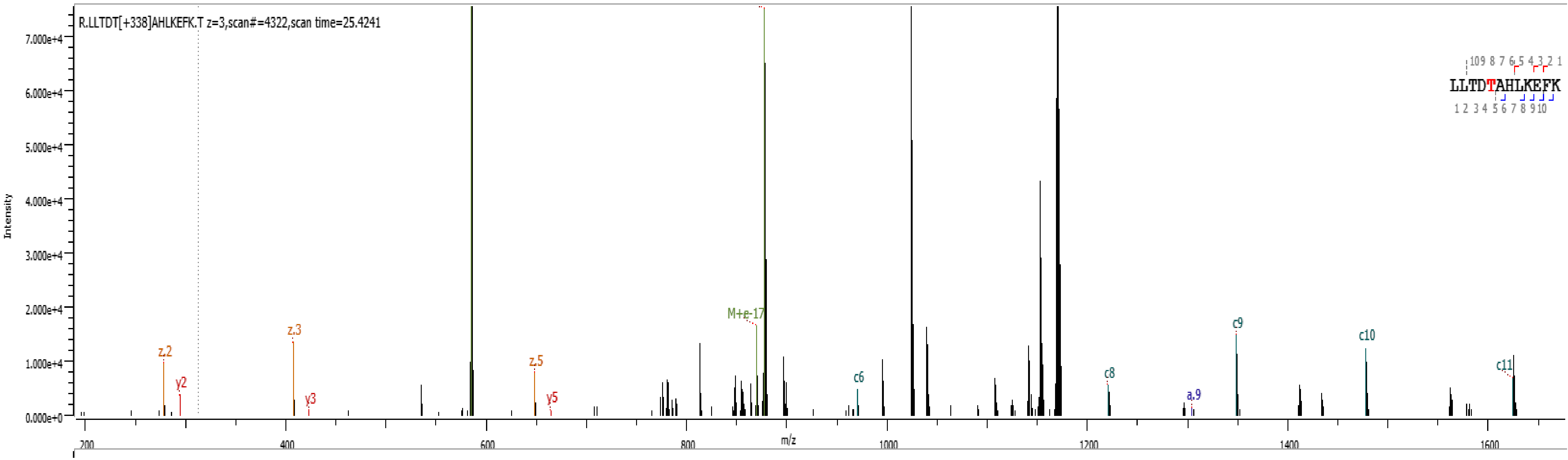
R.LLTDT[+338]AHLKEFK.T z=3,scan#=4322,scan time=25.4241
Intensity
m/z
LLTDTAHLKEFK
z.2
y2
z.3
y3
z.5
y5
M+e-17
c6
c8
a.9
c9
c10
c11

Y

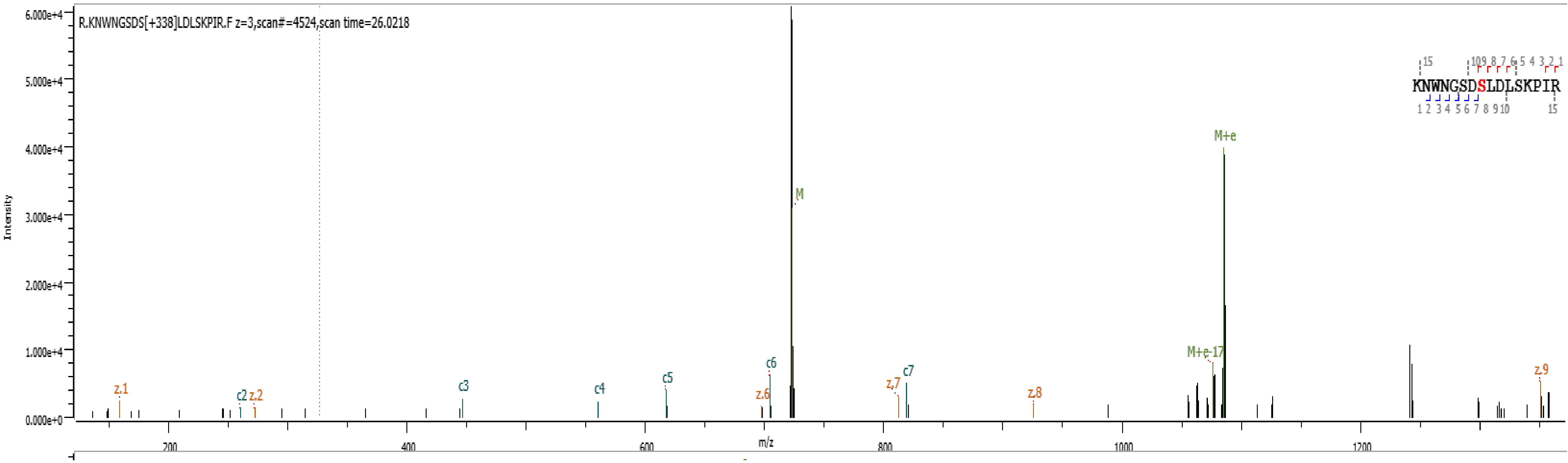
R.KNWNGSDS[+338]LDLSKPIR.F z=3,scan#=4524,scan time=26.0218
Intensity
m/z
KNWNGSDSLDLSKPIR
M
M+e
M+e-17
c2
c3
c4
c5
c6
c7
z.1
z.2
z.6
z.7
z.8
z.9

# Z

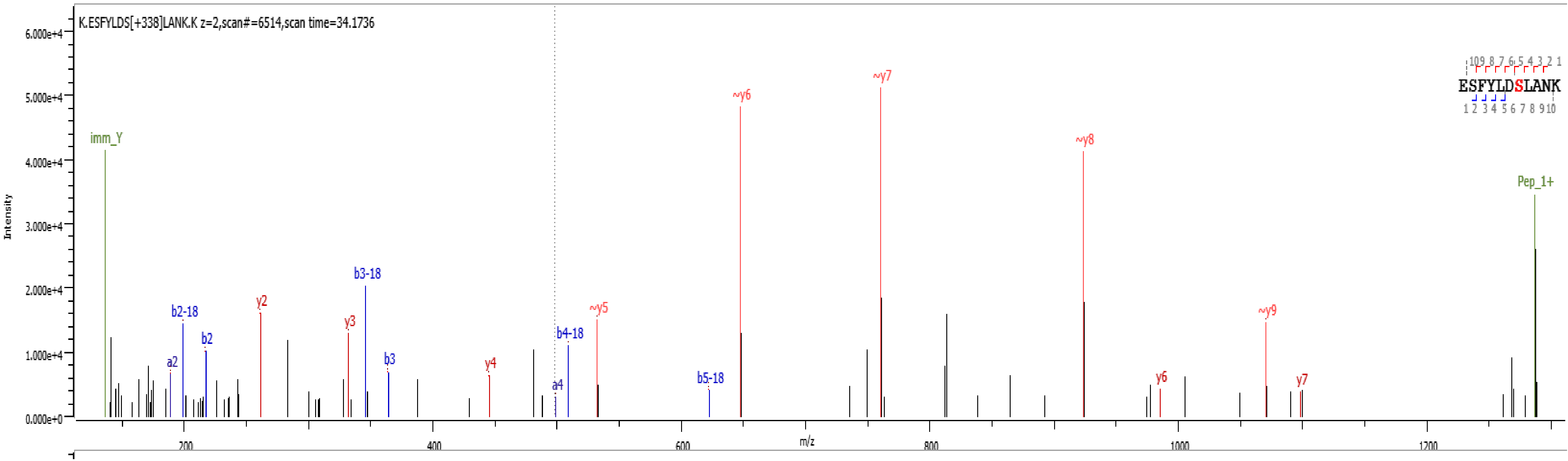

K.ESFYLDS[+338]LANK.K z=2,scan#=6514,scan time=34.1736
Intensity
m/z
imm_Y
a2
b2-18
b2
y2
y3
b3-18
b3
y4
a4
b4-18
~y5
b5-18
~y6
~y7
~y8
y6
~y9
y7
Pep_1+
ESFYLDSLANK

# AA

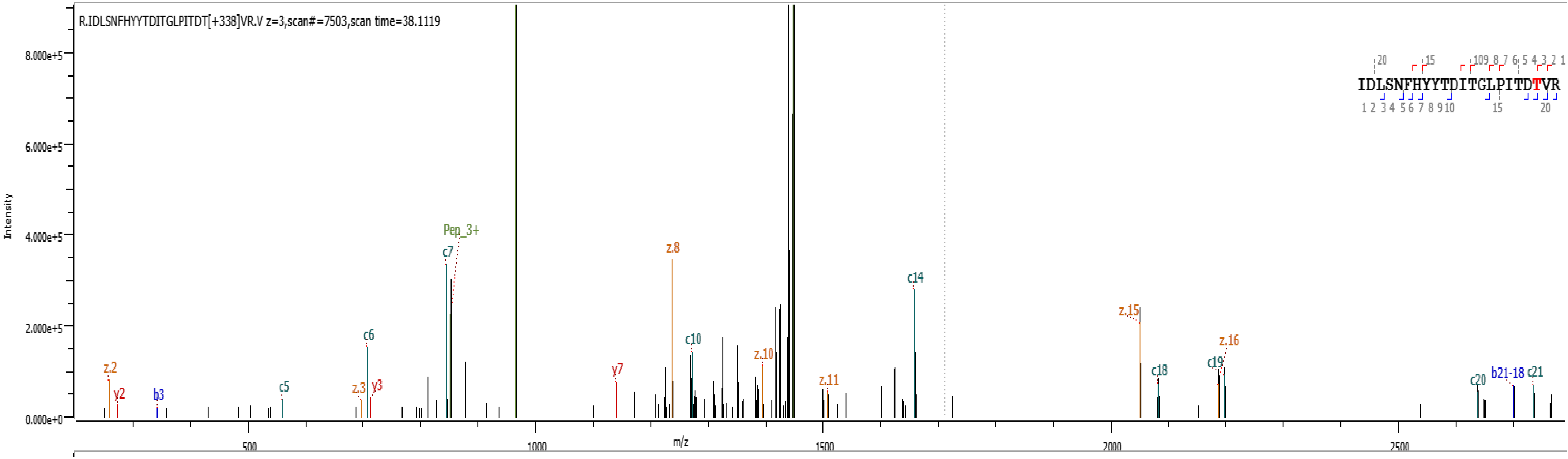
R.IDLSNFHYYTDITGLPITDT[+338]VR.V z=3,scan#=7503,scan time=38.1119
Intensity
m/z
IDLSNFHYYTDITGLPITDTVR
Pep_3+
z.8
c14
z.15
z.16
c10
z.10
c6
c7
y7
c19
c18
c20
b21-18
c21
z.11
z.2
y2
b3
c5
z.3
y3

# AB

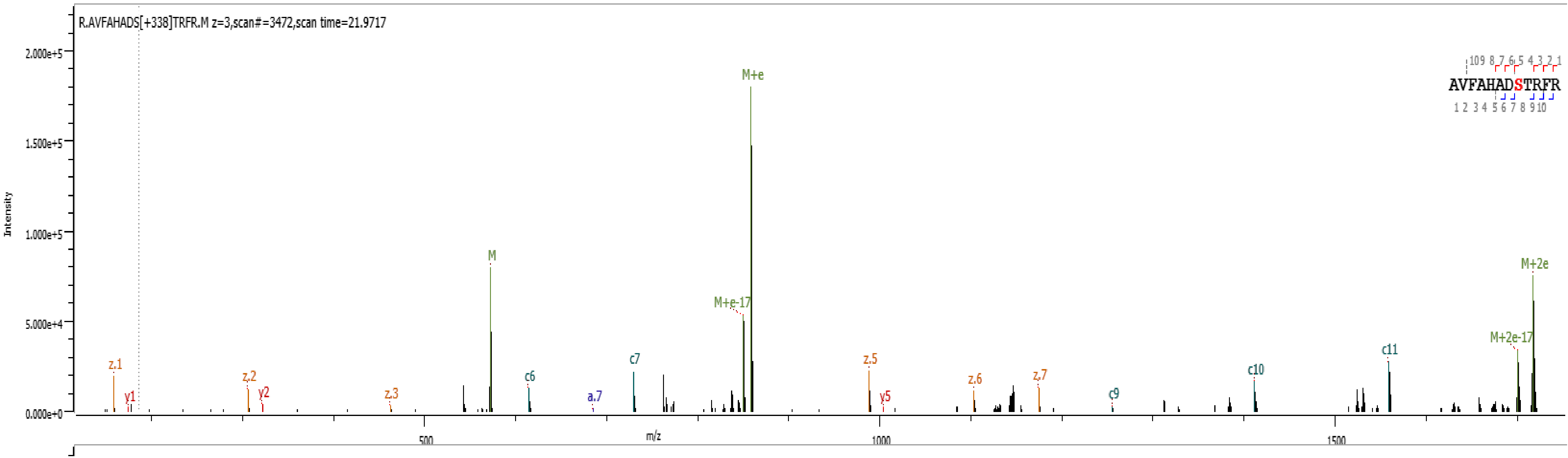
R.AVFAHADS[+338]TRFR.M z=3,scan#=3472,scan time=21.9717
Intensity
2.000e+5
1.500e+5
1.000e+5
5.000e+4
0.000e+0
m/z
500
1000
1500
M+e
M
M+e-17
M+2e
M+2e-17
z.1
y1
z.2
y2
z.3
c6
a.7
c7
z.5
y5
z.6
z.7
c9
c10
c11
AVFAHADSTRFR

# AC

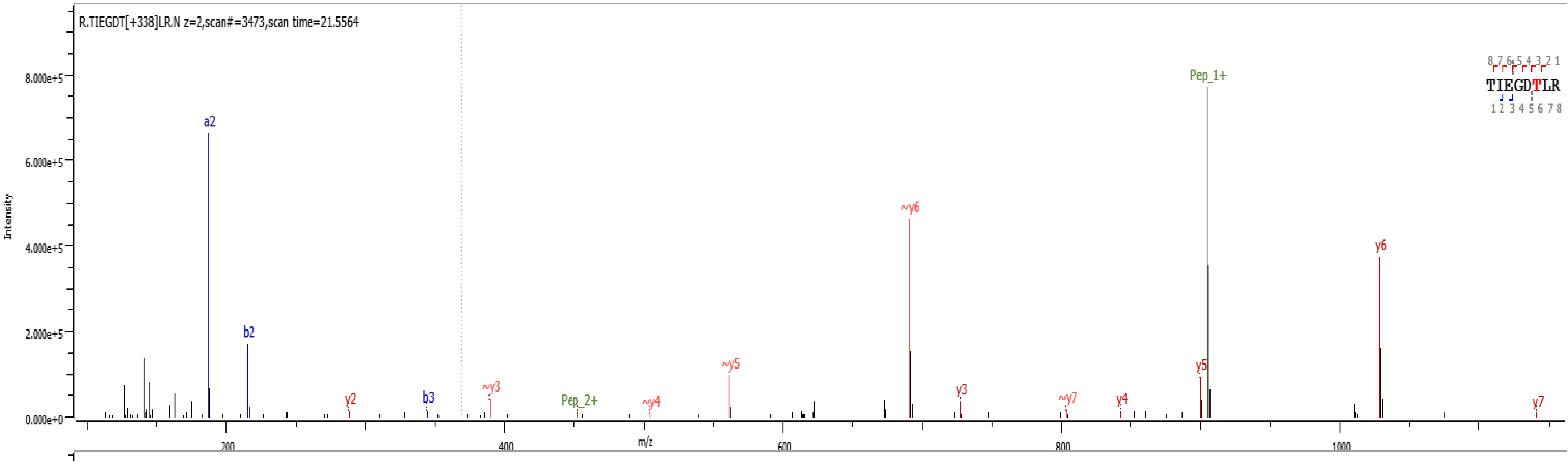

R.TIEGDT[+338]LR.N z=2,scan#=3473,scan time=21.5564
Intensity
8.000e+5
6.000e+5
4.000e+5
2.000e+5
0.000e+0
m/z
200
400
600
800
1000
a2
b2
y2
b3
~y3
Pep_2+
~y4
~y5
~y6
y3
~y7
y4
y5
Pep_1+
y6
y7
8 7 6 5 4 3 2 1
TIEGDTLR
1 2 3 4 5 6 7 8

# AD

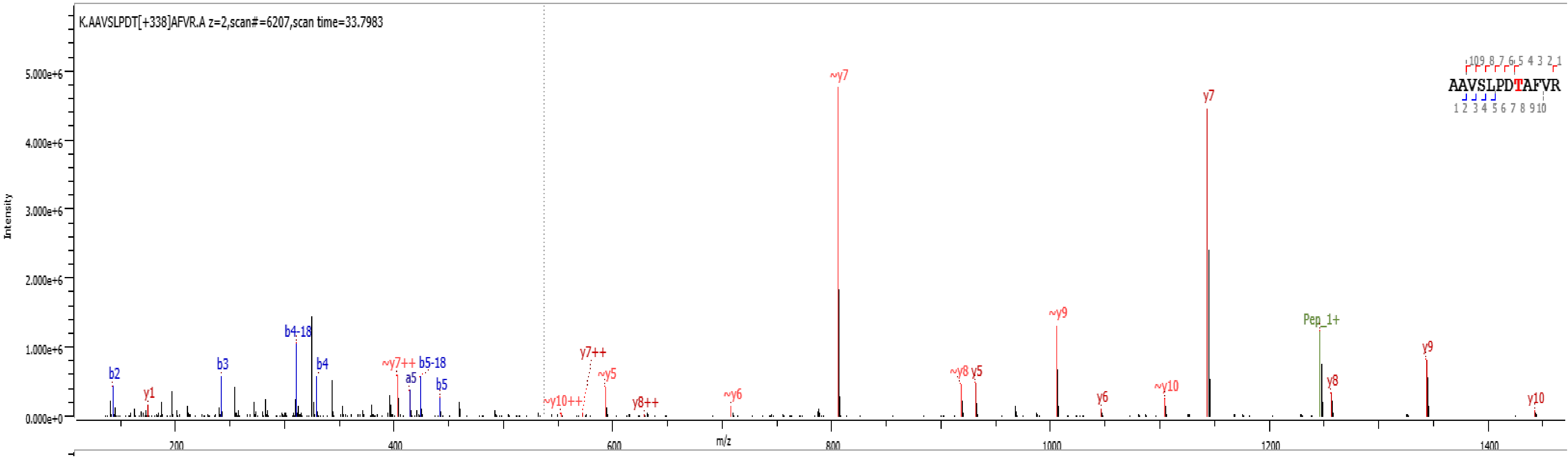
K.AAVSLPDT[+338]AFVR.A z=2,scan#=6207,scan time=33.7983
Intensity
m/z
AAVSLPDTAFVR
~y7
y7
~y9
Pep_1+
y9
y10
b2
y1
b3
b4-18
b4
~y7++
a5
b5-18
b5
~y10++
y7++
~y5
y8++
~y6
~y8
y5
y6
~y10
y8
