# Supplementary material for: Characterization of the O-Glycoproteome of Tannerella forsythia
Source: mSphere. 2021 Sep 15;6(5):e00649-21. doi: 10.1128/mSphere.00649-21 (PMC8550257; doi:10.1128/mSphere.00649-21)
Supplement: FIG S4 [file msphere.00649-21-sf004.docx]

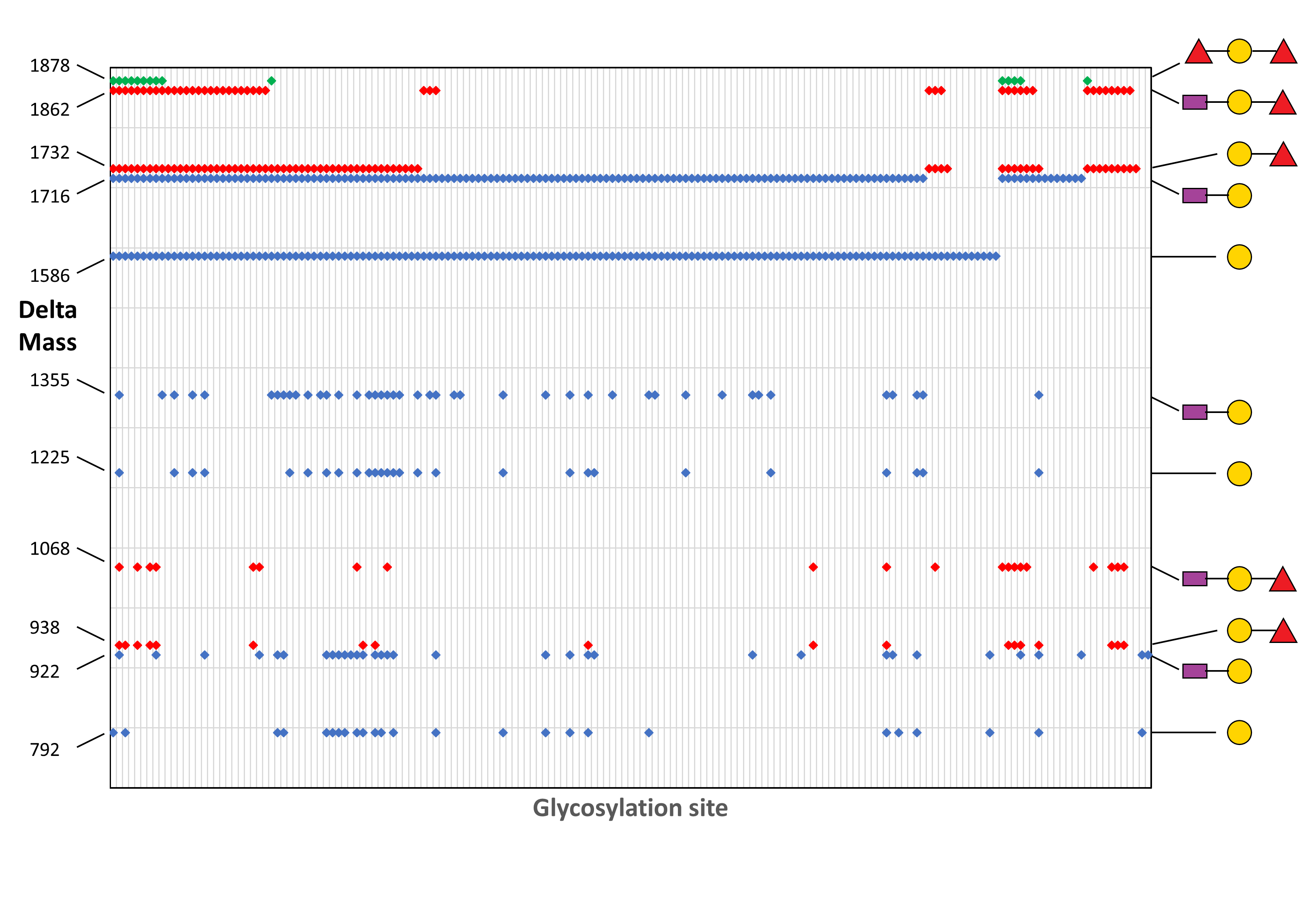


**Fig S4. Correlation of glycoforms according to fucose level**. Each horizontal line of data represents a single glycoform (single Δmass) while each vertical line is a different glycosylation site. It can be seen that the data colored blue correlate and correspond to glycans with no fucose substituent on the Gal while data colored red correlate and correspond to glycans with one fucose substituent on the Gal.
